# A normative model of peripersonal space encoding as performing impact prediction

**DOI:** 10.1371/journal.pcbi.1010464

**Published:** 2022-09-14

**Authors:** Zdenek Straka, Jean-Paul Noel, Matej Hoffmann

**Affiliations:** 1 Department of Cybernetics, Faculty of Electrical Engineering, Czech Technical University in Prague, Prague, Czech Republic; 2 Center for Neural Science, New York University, New York City, New York, United States of America; Karolinska Institutet, SWEDEN

## Abstract

Accurately predicting contact between our bodies and environmental objects is paramount to our evolutionary survival. It has been hypothesized that multisensory neurons responding both to touch on the body, and to auditory or visual stimuli occurring near them—thus delineating our peripersonal space (PPS)—may be a critical player in this computation. However, we lack a normative account (i.e., a model specifying how we *ought to* compute) linking impact prediction and PPS encoding. Here, we leverage Bayesian Decision Theory to develop such a model and show that it recapitulates many of the characteristics of PPS. Namely, a normative model of impact prediction (i) delineates a graded boundary between near and far space, (ii) demonstrates an enlargement of PPS as the speed of incoming stimuli increases, (iii) shows stronger contact prediction for looming than receding stimuli—but critically is still present for receding stimuli when observation uncertainty is non-zero—, (iv) scales with the value we attribute to environmental objects, and finally (v) can account for the differing sizes of PPS for different body parts. Together, these modeling results support the conjecture that PPS reflects the computation of impact prediction, and make a number of testable predictions for future empirical studies.

## Author summary

The brain has neurons that respond to touch on the body, as well as to auditory or visual stimuli occurring near the body. These neurons delineate a graded boundary between the near and far space. Here, we aim at understanding whether the function of these neurons is to predict future impact between the environment and body. To do so, we build a mathematical model that is statistically optimal at predicting future impact, taking into account the costs incurred by an impending collision. Then we examine if its properties are similar to those of the above-mentioned neurons. We find that the model (i) differentiates between the near and far space in a graded fashion, predicts different near/far boundary depths for different (ii) body parts, (iii) object speeds and (iv) directions, and (v) that this boundary scales with the value we attribute to environmental objects. These properties have all been described in behavioral studies and ascribed to neurons responding to objects near the body. Together, these findings suggest *why* the brain has neurons that respond only to objects near the body: to compute predictions of impact.

## Introduction

Predicting environmental impact on our body is a critical computation promoting our evolutionary survival. Interactions between our body and the environment occur within the theater of our peripersonal space (PPS; [1, 2]), the space immediately adjacent to and surrounding our body. In turn, the brain has a specialized fronto-parietal circuit representing multisensory objects and events in a body-centered reference frame when these are near the body [3–5]. There is strong experimental evidence demonstrating that PPS plays a key role in defensive behaviors (see [6] for a seminal review) and initial evidence likewise suggests that PPS encoding plays a role in impact prediction [4, 7, 8]. For instance, stimuli looming toward the body enhance tactile sensitivity at the spatial and temporal location where observers expect impact to occur [9], and PPS enlarges as the speed of incoming stimuli grows [10]. However, we lack a normative account linking impact prediction and PPS.

Modeling efforts have accounted for a number of different aspects of PPS. Magosso and colleagues first introduced a biologically motivated neural network of PPS [11, 12]. This model inherits much of its ability to distinguish between near and far spaces from its local connectivity patterns within unisensory areas. Variants of this model can account for PPS re-sizing after tool use [12, 13], as well as its remapping as a function of the speed of approaching stimuli [14] and recent stimuli statistics [15]. This model may also account for the inflexibility of PPS remapping in autism [16]. Similarly, Bertoni et al. [17] developed a neural network model of PPS, with the innovation that this latter one learns the statistical regularities between visual, tactile, and proprioceptive inputs in order to construct a representation of PPS. In doing so, Bertoni et al.’s model shows how PPS neurons may be anchored to body parts. Straka and Hoffmann [18] have trained a neural network to integrate seen object position and velocity, as well as to predict future tactile contact. However, this model’s predictions of tactile activation, and thus impact, were trained in a supervised manner and the model did not explicitly calculate the probability of future tactile contact. Roncone et al. [19] proposed a PPS model which was trained using a humanoid robot, by nearing objects. The model estimated the likelihood of future contact and used this prediction for avoidance behavior. Perhaps most related to our model, Bufacchi et al. [20] used a 3D geometric model of defensive PPS to fit hand-blink reflex data, assuming uncertainty about stimulus direction in all 3 dimensions and an infinite time-limit.

These models have certainly advanced our understanding of PPS, but share a common limitation in being non-normative. That is, they suggest how PPS and impact prediction could be computed or learned from observations, as opposed to how it ought to be computed. Instead, a wealth of evidence, across a wide variety of fields and tasks (e.g., [21–24]), have shown that humans perceive and perform decisions (near) optimally. Thus, mechanistic models (e.g., neural networks) and human performance should be benchmarked against statistical optimality. Similarly, a strong test of the hypothesis that a functional role of PPS is to perform impact prediction [4, 8] is to build a normative model of the latter, and then contrast the behavior of this model to known properties of PPS encoding.

Here, we use Bayesian Decision Theory [25–28] to propose a normative model of PPS as performing prediction of impact which minimizes the loss/cost such an impact may incur to the agent. We show that this normative model (i) delineates a graded boundary between near and far space [3], (ii) demonstrates a larger PPS as the speed of incoming stimuli increases [10, 14], (iii) shows stronger contact prediction for looming than receding stimuli—but critically is still present for receding stimuli [6, 29, 30]—, (iv) scales with the values of objects (e.g., innocuous vs. potentially dangerous; [31, 32]), and finally (v) can account for differing sizes of PPS for different body parts [33]. Together, these results recapitulate a set of important features of PPS and support the hypothesis that PPS neurons perform contact prediction.

## Results

**Fig 1:**
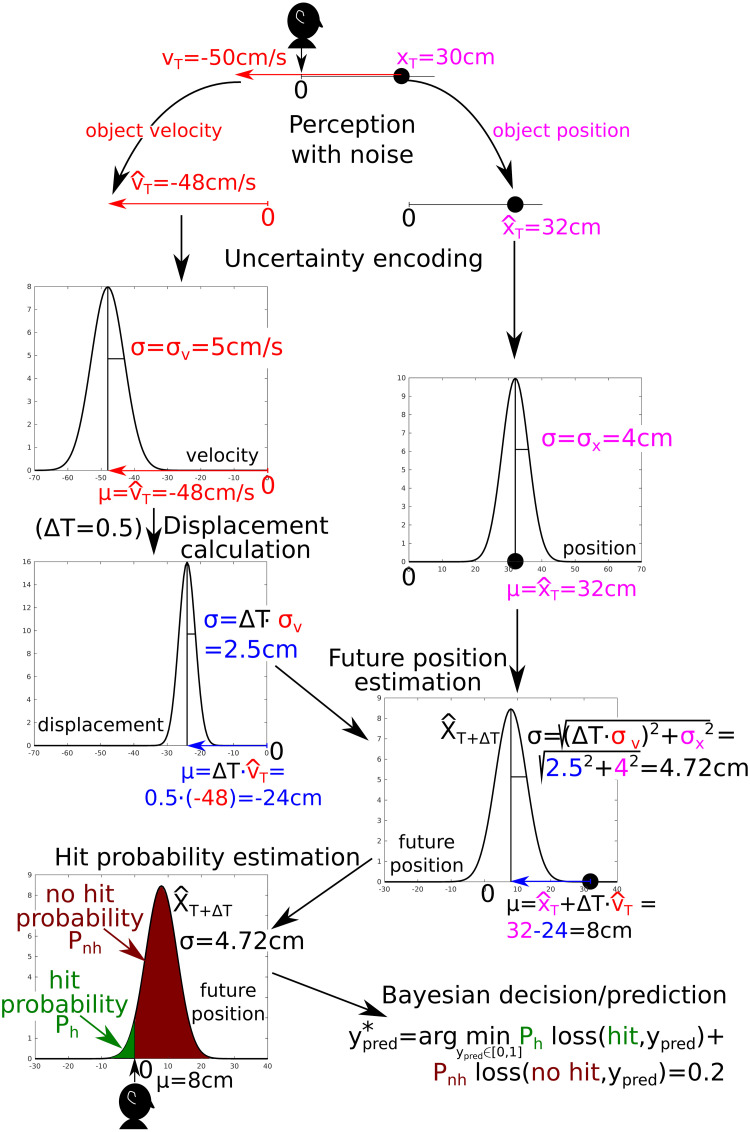
Schema and illustrative example of the contact prediction model. Say an object (black circle) is *x*_*T*_ = 30cm from the body (black head) and is approaching with velocity *v*_*T*_ = −50*cm*/*s*. **Perception with noise**. The nervous system estimates the position and velocity of the object with respect to our body with a given uncertainty. For instance, we may estimate ${\hat{x}}_{T}=32cm$ and ${\hat{v}}_{T}=-48cm/s$. Assuming that the noise is Gaussian, the values ${\hat{x}}_{T},{\hat{v}}_{T}$ are samples from normal distributions *N*(*μ* = *x*_*T*_, *σ*_*x*_), *N*(*μ* = *v*_*T*_, *σ*_*v*_), where *σ*_*x*_ (here, for illustration *σ*_*x*_ = 4*cm*), *σ*_*v*_ (here *σ*_*v*_ = 5*cm*/*s*) reflect the level of noise. Further, we assume the brain encodes not only point estimates (${\hat{x}}_{T},{\hat{v}}_{T}$), but also their uncertainty—the estimates are encoded as normal distributions $N(\mu ={\hat{x}}_{T},\sigma =\sigma_{x})$ and $N(\mu ={\hat{v}}_{T},\sigma =\sigma_{v})$, respectively (see Derivation of the normative impact prediction model for details). **Displacement calculation**. According to Δ*T*, the object displacement distribution is $N(\mu =\Delta T\cdot {\hat{v}}_{T},\sigma =\Delta T\cdot \sigma_{v})$. **Future position estimation**. Knowing the current position and displacement during Δ*T*, the position at time *T* + Δ*T* is calculated as *position*_*T*+Δ*T*_ = *position*_*T*_ + *displacement*. Consequently, the distribution of possible future positions ${\hat{X}}_{T+\Delta T}$ is $N(\mu ={\hat{x}}_{T}+\Delta T\cdot {\hat{v}}_{T},\sigma =\sqrt{{\left(\Delta T\cdot \sigma_{v}\right)}^{2}+\sigma_{x}^{2}})$. **Hit probability estimation**. As the body position is at *x* = 0, the object will hit the body if its position is equal or smaller than zero (see the green part of the distribution). Therefore, the estimated probability of body hit (i.e., *y* = 1) is $P\left(y=1|\left({\hat{x}}_{T},\sigma_{x}\right),\left({\hat{v}}_{T},\sigma_{v}\right)\right)=P\left({\hat{X}}_{T+\Delta T}\leq 0\right)$. The probability estimation of no contact is $P\left(y=0|\left({\hat{x}}_{T},\sigma_{x}\right),\left({\hat{v}}_{T},\sigma_{v}\right)\right)=1-P\left(y=1|\left({\hat{x}}_{T},\sigma_{x}\right),\left({\hat{v}}_{T},\sigma_{v}\right)\right)$, which corresponds to the crimson part of the distribution. **Bayesian decision/prediction**. Following Eq (1), a prediction $y_{pred}^{*}$—which minimizes the expected loss—is calculated. See S1 and S2 Files for details of the computation.

We developed a Bayesian observer inferring whether contact between an external object and the body would occur within the next time step. An overview of the model is given in Fig 1 and S1 File (for full detail see the Materials and methods section). Briefly, at time *T*, an object has position *x*_*T*_ and moves with velocity *v*_*T*_. The observer is tasked with predicting whether at or before *T* + Δ*T* this object will make contact with the body. This prediction takes into account two components. First, the probability estimation of the object making contact with the body, given its perceived position and velocity, including its uncertainty. Second, the loss (i.e., penalty) incurred if the prediction is incorrect. We denote the possible impact of the object on the body as *y* ∈ {0, 1}, which is a binary variable—either there is contact with the body or there is not. Instead, *y*_*pred*_ ∈ [0, 1], a continuous value, is the prediction whether contact will occur or not, taking into account the estimation of probability of contact and the loss function. Optimal impact prediction is denoted by $y_{pred}^{*}$.

According to Bayesian Decision Theory (see e.g., [25, 26]) the optimal decision—in our case the impact prediction $y_{pred}^{*}$—is

$$\begin{array}{c} y_{pred}^{*}=\text{arg}\underset{y_{pred}\in \left[0,1\right]}{\text{min}}L\left(\left({\hat{x}}_{T},\sigma_{x}\right),\left({\hat{v}}_{T},\sigma_{v}\right),y_{pred}\right) \end{array}$$ (1)

where

$$\begin{array}{c} L\left(\left({\hat{x}}_{T},\sigma_{x}\right),\left({\hat{v}}_{T},\sigma_{v}\right),y_{pred}\right)=P\left(y=1|\left({\hat{x}}_{T},\sigma_{x}\right),\left({\hat{v}}_{T},\sigma_{v}\right)\right)\cdot loss\left(y=1,y_{pred}\right)+\\ P\left(y=0|\left({\hat{x}}_{T},\sigma_{x}\right),\left({\hat{v}}_{T},\sigma_{v}\right)\right)\cdot loss\left(y=0,y_{pred}\right) \end{array}$$ (2)

and ${\hat{x}}_{T},{\hat{v}}_{T}$ are respectively the observer’s point estimates of the object position *x*_*T*_ and velocity *v*_*T*_ at time *T* (see Fig 1). The estimates need not be the same as the actual object position and velocity, given that perception may be distorted by observation noise (see Derivation of the normative impact prediction model for details). Uncertainty about the position and velocity are respectively expressed by *σ*_*x*_, *σ*_*v*_. Stimuli perceived less accurately (e.g., visual stimuli at low contrast, or auditory localization as opposed to visual localization) result in greater *σ*_*x*_ and *σ*_*v*_. To include this uncertainty, position and velocity estimates are respectively encoded as normal distributions $N(\mu ={\hat{x}}_{T},\sigma =\sigma_{x})$ and $N(\mu ={\hat{v}}_{T},\sigma =\sigma_{v})$. Displacement of the object during Δ*T* is encoded as normal distribution $N(\mu =\Delta T\cdot {\hat{v}}_{T},\sigma =\Delta T\cdot \sigma_{v})$ (see Fig 1 or Derivation of the normative impact prediction model for details).

Merging the position and displacement estimations, the probability $P(y|\left({\hat{x}}_{T},\sigma_{x}\right),\left({\hat{v}}_{T},\sigma_{v}\right))$ of the external object making contact with the body (*y* = 1) at or before *T* + Δ*T* given the agent’s observations at time *T* is estimated (see the calculation in Fig 1 or in Derivation of the normative impact prediction model). Conversely, the estimated probability that the external object will not make impact with the body is $P\left(y=0|\left({\hat{x}}_{T},\sigma_{x}\right),\left({\hat{v}}_{T},\sigma_{v}\right)\right)=1-P\left(y=1|\left({\hat{x}}_{T},\sigma_{x}\right),\left({\hat{v}}_{T},\sigma_{v}\right)\right)$.

The second important component in computing the value associated with an object’s velocity and distance to the body is the utility function, loss(*y*, *y*_*pred*_). For a predicted value *y*_*pred*_, it enables to calculate the corresponding loss associated with *y* ∈ {0, 1}. For a zero-one loss function—loss is 0 if the prediction *y*_*pred*_ equals *y*, 1 otherwise—the optimal prediction (i.e., minimizing expected loss) is to predict the state with the highest probability. More generally, however, a number of different loss functions could be used. Here, we define a fairly general loss function as,

$$\begin{array}{c} loss\left(y,y_{pred}\right)=FP\;\text{max}\;{\left(0,y_{pred}-y\right)}^{2}+FN\;\text{max}\;{\left(0,y-y_{pred}\right)}^{2}, \end{array}$$ (3)

where *FP, FN* ∈ [0, ∞] are respectively the false positive and false negative factors, and max(0, *x*) is a function which outputs *x* for *x* ≥ 0 and 0 for *x* < 0. In other words, FP determines the penalty, or cost, associated with predicting impact when none occurs, and FN determines the penalty associated with not predicting impact when one does occur.

Throughout the article, we typically assume *FN* > *FP*, as we focus on defensive PPS and given that it is arguably better to erroneously predict tactile activation (FP) than it is to experience impact on our bodies without predicting it (FN) (see *The Precautionary Principle*). In this case an impact prediction minimizing the expected loss is performed. We typically use *FN* = 5;*FP* = 1. This choice is arbitrary and was chosen experimentally. The effect of different choices (1, 5, 100) is illustrated in Section A graded PPS “boundary”—Effect of sensory uncertainty and cost of false negative prediction. We did not study the case where *FN* < *FP*, which may correspond to appetitive actions like reaching or grasping (see also [34]), but such values can be readily tested with the current model. Furthermore, for the special case when *FP* = *FN*, the model performs *optimal impact prediction*—the error between the prediction and the actual state is minimized. In this case, the optimal prediction is equal to the hit probability estimation. In what follows, we complement every graph in the main body of the article (with *FN* = 5;*FP* = 1) with a twin figure in the S1–S5 Figs where *FN* = *FP* = 1.

Putting the above together (estimated probability of touch and loss function), we may write the full expression (see Eq (6) for the derivation),

$$\begin{array}{c} L\left(\left({\hat{x}}_{T},\sigma_{x}\right),\left({\hat{v}}_{T},\sigma_{v}\right),y_{pred}\right)=P\left(y=1|\left({\hat{x}}_{T},\sigma_{x}\right),\left({\hat{v}}_{T},\sigma_{v}\right)\right)\cdot loss\left(y=1,y_{pred}\right)+\\ P\left(y=0|\left({\hat{x}}_{T},\sigma_{x}\right),\left({\hat{v}}_{T},\sigma_{v}\right)\right)\cdot loss\left(y=0,y_{pred}\right)=\\ P\left(y=1|\left({\hat{x}}_{T},\sigma_{x}\right),\left({\hat{v}}_{T},\sigma_{v}\right)\right)FN{\left(1-y_{pred}\right)}^{2}+(1-P\left(y=1|\left({\hat{x}}_{T},\sigma_{x}\right),\left({\hat{v}}_{T},\sigma_{v}\right)\right)FPy_{pred}^{2} \end{array}$$ (4)

In what follows, we perform simulations to compare properties of this normative model of impact prediction with known properties of PPS encoding.

### A graded PPS “boundary”—Effect of sensory uncertainty and cost of false negative prediction

The study of PPS was jump-started by the realization that the primate brain has a set of neurons encoding multisensory objects when these are near from the body [2, 6, 10, 30, 35, 36]. Thus, first and foremost, if the impact prediction model accounts for PPS, it ought to differentiate between near and far spaces. In addition, more recently authors have highlighted that this PPS “boundary” is not all-or-none, but graded [37]. Thus, in a second step we question if and how the impact prediction model allows for graded PPS “boundaries”.

First, we build a baseline model with the parameter values listed in Table 1.

**Table 1:** Baseline model parameters. Negative values for velocity *v*_*T*_ indicate objects approaching the body, while positive values would indicate objects receding from the body. In simulations we manipulate each of these parameters, except for *σ*_*x*_ and *FP*.

velocity	*v*_*T*_ = −25*cm*/*s*
velocity estimation uncertainty	*σ*_*v*_ = 20*cm*/*s*
position estimation uncertainty	*σ*_*x*_ = 2.5*cm*
false negative factor	*FN* = 5
false positive factor	*FP* = 1
prediction time step	Δ*T* = 0.5*s*

**Fig 2:**
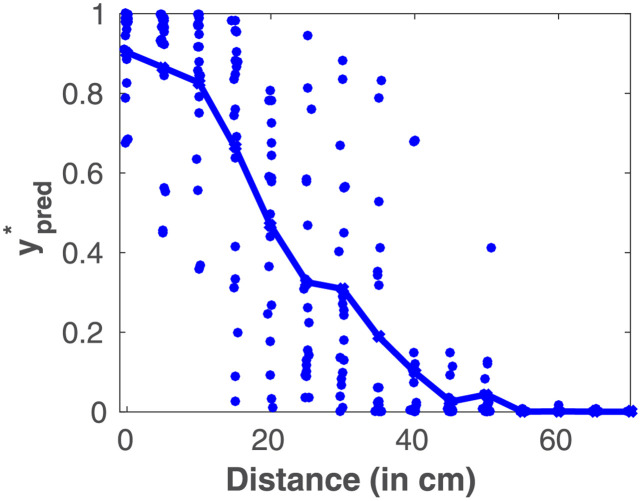
PPS as optimal impact utility prediction for baseline parameters. Blue dots—20 for each distance—are individual predictions (samples) of $y_{pred}^{*}$. Blue line—mean of 20 repetitions. Parameters used are in Table 1. See S1 Fig for a version with *FN* = *FP* = 1.

As shown in Fig 2, the model generates predictions of contact $y_{pred}^{*}$ that grow gradually with object proximity to the body. Further, it differentiates between a “far space” where touch is not likely to occur, and a “near space” where touch is highly likely to occur. If we consider the PPS “boundary” as the first value of predicted impact where $mean(y_{pred}^{*})>0.01$ (see [14], Fig 17 & 18 for a similar approach). With this basal configuration the impact prediction model specifies a “boundary” between far and near space at about 50cm from the body.

**Fig 3:**
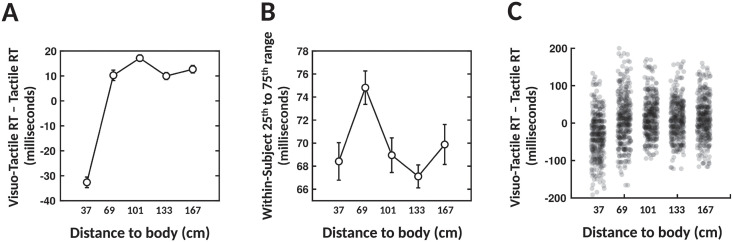
Variability in multisensory facilitation as a function of distance from the self–empirical data. New evaluation of data from Masson et al. [39]. (A) Visuo-tactile facilitation of reaction times (RT) as a function of distance to the body—means and standard errors across subjects. (B) Within-subject variability of reaction times. (C) Aggregate subject, combining visuo-tactile RT facilitation across all subjects.

An alternative operationalization of the PPS “boundary” used in the literature is the midpoint of a sigmoid function (e.g., [29, 33, 38]). Interestingly, close examination not solely of the mean response (solid line), but also of the variability (blue dots) with the model (Fig 2) seems to indicate that impact prediction estimates are most variable near the PPS “boundary” region. We examined if this property was apparent in empirical data by re-analyzing data from [39]. In this study, human observers (n = 19) were asked to respond to touch as quickly as possible as task irrelevant visual stimuli approached their body in virtual reality. In Fig 3A we show that reaction times to visuo-tactile stimuli were faster than to tactile stimuli alone. Further, this multisensory facilitation was most apparent as visual stimuli were near the body—indexing the encoding of PPS. In this dataset, the PPS “boundary” was located between the first and second visuo-tactile distance indexed. Most importantly, in Fig 3B we quantified variability in reaction times, at a single subject level. That is, while reports (e.g., [15, 16, 40, 41]) typically illustrate between-subject variability (for instance by showing standard errors of the mean across subjects), there is no quantification of within-subject variability. Here, for each subject we measure the range between the 25th and 75th percentile of their reaction times, for a given subject and distance. Fig 3B depicts the mean of these ranges across subjects, and shows that within-subject variability peaked at the second distance indexed. In Fig 3C we show all reaction times measured, again showing the largest range at the second distance index. Altogether, the empirical results concur with the modeling prediction that within-subject variability is largest near the PPS “boundary”.

Next, we questioned if and how this model may account for steepness in the PPS boundary, as well as for changes in its size—the most common experimental finding (e.g., PPS expanding with tool use [42], or during walking [40], or bodily illusions [41]). Conveniently, this normative model of impact prediction in essence has two degrees of freedom: (1) the uncertainty associated with perceptual observations, and (2) the ratio of *FP, FN*, dictating an appraisal of the danger associated with the objects approaching the body. For simplicity, we refer to these degrees of freedom respectively as a ‘sensory’ and ‘cognitive’ node, yet it is well established that socio-emotional contexts and motor constraints/possibilities impact our appraisal of the value of objects in our environment (e.g., see [4, 5, 37]). One additional parameter is the Δ*T*. This is the prediction time step of the model—a time interval for which contact estimation is performed. The object may hit the body at any moment within this interval. Its effects will be studied in Section PPS shape modulated by prediction time step. The rest of parameters (e.g., *x*_*T*_, *v*_*T*_) depend on the physical state of the world.

**Fig 4:**
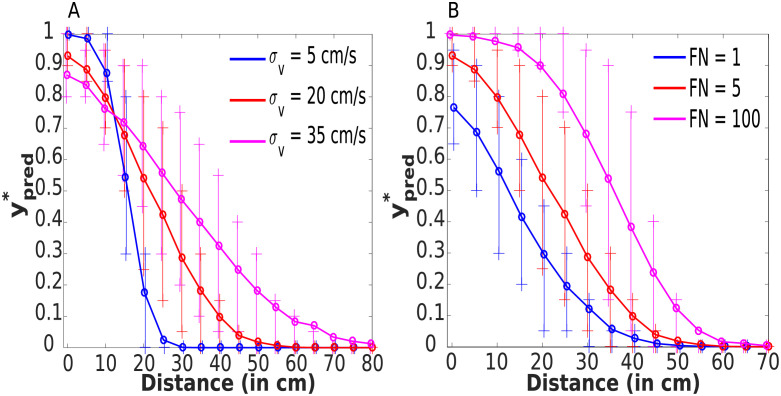
Effect of stimulus uncertainty and the False Negative (FN) penalty parameters. Dependency between the mean of 1000 predicted tactile activations $y_{pred}^{*}$ (for each distance) and distance *x*_*T*_ (in centimeters) of the stimuli from the body. The symbols “+” indicate 25th and 75th percentiles which are calculated from 1000 predicted values $y_{pred}^{*}$ for each distance. **(A) The size of PPS and slope of its boundary are modulated by *σ*_*v*_**. **(B) The size of PPS, but only minimally the slope of its boundary, are modulated by *FN***. Parameters used are in Table 1 (except for *σ*_*v*_ in (A) and *FN* in (B)). See S2 Fig—the right upper panel—for a version of subfigure **A** with *FN* = *FP* = 1.

In turn, in Fig 4A and 4B we respectively manipulate *σ*_*v*_ (5, 20, and 35 cm/s) and *FN* (1, 5, and 100). As shown in Fig 4A, changes in sensory uncertainty lead to concurrent increase in PPS size (i.e., the first distance at which $y_{pred}^{*}$ is higher than 0.01 being farther and farther in space), and a decrease in the sharpness of its boundary. On the other hand, increasing *FN* (while maintaining *FP* constant at 1), Fig 4B, increases the size of PPS while leaving the shape of its boundary virtually unchanged. Together, these results demonstrate that the normative model of impact prediction not only differentiates between a near and far space but also shows that both sensory and higher-level value attributes [37] may impact the size and shape of PPS. In S6 Fig we explore how *σ*_*v*_, Δ*T* and *FN* may simultaneously impact the gradient of the PPS boundary and PPS size.

Finally, note that the observed effect that increasing perceptual uncertainty increases the PPS size is apparent when the PPS boundary is operationalized as the farthest distance for which $mean(y_{pred}^{*})>0.01$. If instead the midpoint of a sigmoid function is estimated and used as a proxy for PPS size, the effect is significantly smaller. For the special case where *FP* = *FN* = 1, S2 Fig, top panels, there is no effect on “PPS size” at all.

### PPS encoding and object velocity

In addition to defining a graded separation between near and far spaces, PPS encoding is also modulated by the characteristics of nearby external objects, such as their velocity [10, 14], movement direction [6, 29, 30], and valence [31, 32]. In the next three sections we tackle each of these properties in turn.

**Fig 5:**
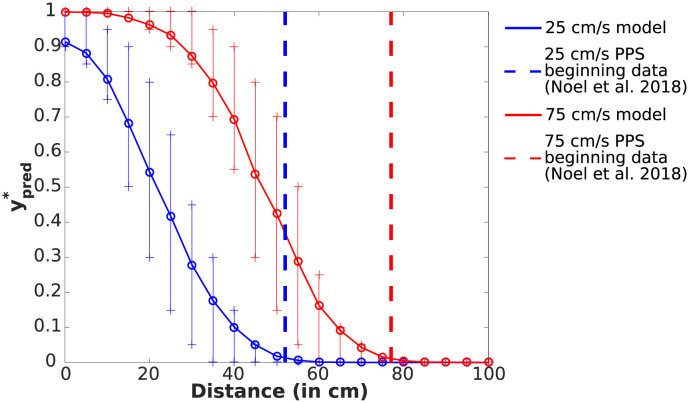
Comparison of PPS sizes for object velocities of -25 and -75 cm/s. Dependency between the mean of 1000 repetitions of impact predictions $y_{pred}^{*}$ and distance *x*_*T*_ (in centimeters) between the stimuli and body, for different object velocities. The symbol “+” indicates 25th and 75th percentiles which are calculated from 1000 predicted values $y_{pred}^{*}$ for each distance. Notice that the beginning of PPS—defined as the farthest distance for which $mean(y_{pred}^{*})>0.01$—roughly corresponds to the PPS beginning around the face determined by [14]. Except for the velocity *v*_*T*_ = −75*cm*/*s*, the baseline parameters from Table 1 are used. See S3 Fig for a version with *FN* = *FP* = 1.

PPS size expands with the increasing velocity of incoming stimuli [10, 14]. Hence, we questioned whether our model recapitulates this finding. The simulation setup mimicked the setting from [14], with an object approaching the observer with a fixed velocity *v*_*T*_ equal to -25 or -75 cm/s (looming toward the subject). As shown in Fig 5, the impact prediction model inherently shows the dependency between distance of the object to the observer and impact prediction $y_{pred}^{*}$ for both velocities. In fact, if we again operationalize the PPS “boundary” as the farthest distance for which $mean(y_{pred}^{*})>0.01$, our simulation roughly corresponds to the size of PPS empirically measured around the face (i.e., 52 cm for 25 cm/s and 77 cm for velocity 75 cm/s; [14]). Thus, while Noel et al. [14] hypothesize that the enlargement of PPS during increasing object velocity is due to neural adaptation (i.e., progressively stronger inputs are needed to drive a neuron that has been active for a given time), here we are agnostic about the neural implementation and instead show that the physics of our environment naturally leads to an enlargement of PPS with increased object velocities under a framework of impact prediction (see [17] for a similar demonstration that PPS encoding results from the physics of the environment wherein touch is more likely to occur when objects are near the body).

### PPS encoding and looming versus receding objects

PPS encoding is also modulated by the movement direction of objects in the external environment. Namely, neurons mapping PPS are most readily driven by looming, as opposed to receding sensory stimuli [6, 30]. Here we replicate this situation by simulating objects moving with negative (toward the body) or positive (away from the body) velocities. Further, to extend on the empirical data and generate predictions for further experiments, we also simulate objects moving at different speeds (*v*_*T*_ = 12.5cm/s or 25cm/s) and with different levels of sensory uncertainty (*σ*_*v*_ = 5cm/s, 20cm/s, or 35cm/s), both while approaching or receding from the observer.

**Fig 6:**
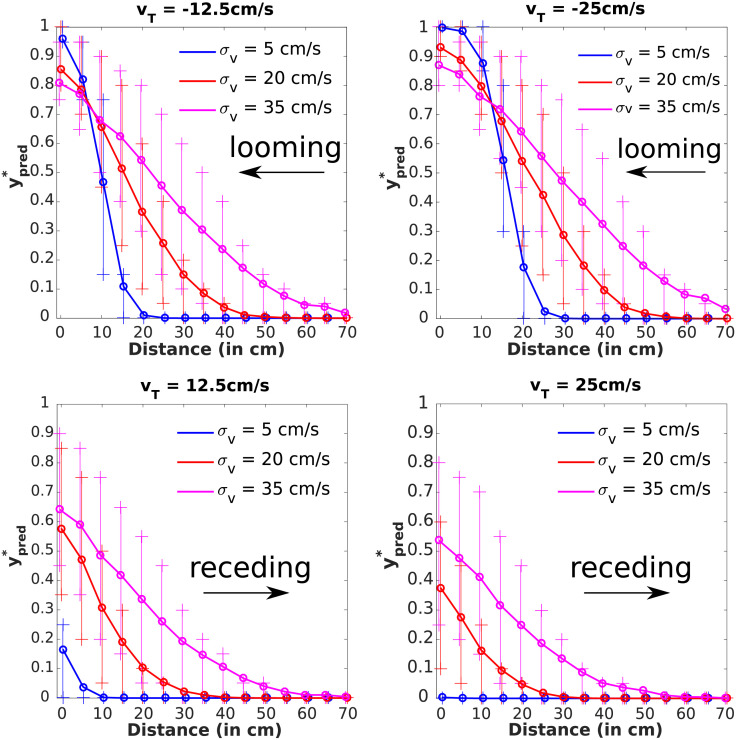
A looming stimulus leads to a higher response than a receding one. The stimulus is looming (receding) to (from) the body with velocity *v*_*T*_ size 12.5 or 25 cm/s. The horizontal axis is the distance *x*_*T*_ of the stimulus from the body. The vertical axis corresponds to the impact prediction $y_{pred}^{*}$—for the mean and 25th/75th percentiles of 1000 predictions for each distance. (**Left column**) The speed of the stimulus was *v*_*T*_ = ±12.5*cm*/*s*. Although the prediction values were significantly smaller for the receding movement, it was still slow enough to get significant impact prediction values even for the receding movement. With increasing velocity uncertainty *σ*_*v*_ of the stimulus, the prediction values increased. (**Right column**) Speed was increased to *v*_*T*_ = ±25*cm*/*s*. This led to reduction of impact prediction values $y_{pred}^{*}$ for the receding movement compared with the smaller speed case. The parameters not listed here take values from Table 1. See S2 Fig for a version with *FN* = *FP* = 1.

As expected, the results demonstrate that when objects loomed toward the body, the predicted tactile activation was higher than when it receded from the body—see Fig 6 and compare the curves corresponding to the same speed *v*_*T*_ and uncertainty *σ*_*v*_ but with opposite directions. Most importantly, our model still generated non-zero $y_{pred}^{*}$ when the object recedes from the body. This is due to object position and velocity estimations having non-zero uncertainties *σ*_*x*_, *σ*_*v*_. Namely, predicted contact for a receding stimulus would be zero if the location and velocity of stimuli were known without any uncertainty (i.e., *σ*_*x*_ and *σ*_*v*_ were zeros). The fact that the current simulations and Bayesian Decision Theory are able to recapitulate not only a response to looming, but also to receding stimuli, supports the hypothesis that PPS reflects a stochastic computation of impact prediction.

Further, we can use this framework to make specific predictions for future empirical work. Namely, according to this model, when looming stimuli increase in speed, PPS expands (see above). However, when receding stimuli increase in speed, there is a negligible probability that at the next time-point the object will make contact with the body (i.e., increased velocity away from the observer offsets the effect of object position being uncertain). Thus, while PPS should expand with increasing velocity of looming stimuli [6, 29, 30], there should be no discernible PPS gradient with fast receding stimuli. Similarly, the ability to delineate a PPS boundary should decrease with increasing sensory uncertainty during looming object trajectories (i.e., the boundary becomes shallower). To the best of our knowledge, these experimental conditions (looming and receding object trajectories during different velocities and uncertainty) have not been tested, and will constitute an important future test in ratifying PPS as predicting future impact.

### PPS encoding and object value

The approach of dangerous objects leads to an expansion of PPS (see e.g., [31, 32, 38, 43]). Within our normative impact prediction model, this effect would *a priori* seem most naturally accommodated by a change in *FN*. However, it may also be argued that greater encoding resources may be attributed to the encoding of dangerous objects, for instance via attentional mechanisms (see [44]), and hence reduce *σ*_*v*_.

As demonstrated above (Fig 4), these competing hypotheses conveniently lead to different predictions. If the expansion of PPS during approach of dangerous objects is due to an increase in *FN* (Fig 4B), we should observe a change in PPS size, with nearly no corresponding change in its gradient. On the other hand, if *σ*_*v*_ decreases (Fig 4A), the PPS “boundary” becomes sharper, and importantly, this leads to shrinking rather than expansion of the size of PPS.

Taffou and Viaud-Delmon [43] used ecological auditory stimuli (dog growling vs. sheep bleating) and reported that PPS expanded in the dog condition, specifically in subjects scared of dogs. They did not explicitly report on the gradient of PPS, yet visual examination suggests no difference between dog and sheep conditions. This—PPS expansion and no apparent change in gradient—putatively suggests that the effect reported in [43] is “cognitive” in nature (i.e., originates from the loss function, *FN*) Importantly, this effect, as interpreted under the current modeling framework also highlights a critical element of the Bayesian observer performing contact prediction; namely that beyond optimizing the prediction of the probability that touch will occur, PPS encoding also ought to optimize the utility associated with impact prediction.

Ferri et al. [38] ratify the conclusion from [43], while also directly comparing ecological and artificial stimuli. In a first experiment, the authors present artificial sounds associated with negative and neutral valence—broadband Brown and White noise, respectively (see [38]). The results show both an expansion and sharpening of PPS during the negative-valence condition. Our model would predict that this may be a simultaneous “sensory” effect driving the change in PPS boundary steepness and a “cognitive” effect driving the PPS expansion and overriding any shrinking due to the new shape of the PPS boundary as a result of decrease in *σ*_*v*_.

Together, this pattern of results highlights the importance in fully characterizing changes in PPS encoding (only when size and gradient are quantified, one can attribute these effects to “sensory” vs. “cognitive” in nature). Further, they suggest that when using ecologically valid sounds—but not artificial stimuli—, enlargements of PPS are most likely due to modulations in the loss function and not low level sensory components. Lastly, these results highlight that, according to the current framework, not all previously reported characteristics of PPS encoding may be explained by either environmental factors or changes in the probability of touch occurring. Instead, impact prediction must also account for the value attributed to environmental objects [37].

### PPS size across different body parts

Beyond defining a graded boundary between near and far space that is modulated by context, another important characteristic of PPS is that it is dependent on body-part, with PPS growing in size from hand to face to torso [33]. The differing size of PPS across body parts is unlikely due to modulations in the sensory uncertainty associated with object position or velocity (*σ*_*x*_ and *σ*_*v*_) given that approaching objects are perceived by exteroception (i.e., vision or audition) which is common across body parts. In theory, the ratio between *FN* and *FP* could account for the different sizes of PPS across body parts, but we would have to posit *FN* being larger for the torso than the face, and it is not immediately clear why this would be the case. Perhaps the most parsimonious explanation would be that the difference in PPS size simply reflects differences in body-part size. In order to test this possibility, we extend the model from 1-dimensional to 3-dimensional. We only model the face and torso in this section.

**Fig 7:**
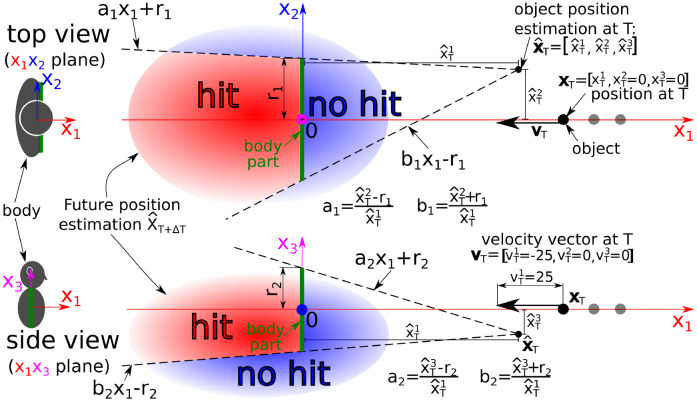
3D experimental scenario. An object is looming to a body part (2D rectangle with size [2 ⋅ *r*_1_, 2 ⋅ *r*_2_] enclosed in 3D space). As the object moves along the *x*_1_ axis, it has position $x_{T}=\left[x_{T}^{1},x_{T}^{2}=0cm,x_{T}^{3}=0cm\right]$ and velocity $v_{T}=\left[v_{T}^{1}=-25cm/s,v_{T}^{2}=0cm/s,v_{T}^{3}=0cm/s\right]$ at time *T*. As the uncertainty in position estimation is nonzero ($\sigma_{x}=\left[\sigma_{x}^{1}>0,\sigma_{x}^{2}>0,\sigma_{x}^{3}>0\right]$), the point position estimation ${\hat{x}}_{T}=\left[{\hat{x}}_{T}^{1},{\hat{x}}_{T}^{2},{\hat{x}}_{T}^{3}\right]$ does not correspond to **x**_*T*_. Future position estimation ${\hat{X}}_{T+\Delta T}$ with a multivariate normal distribution is then calculated (see Section Extension to 3D space for details). The red area of ${\hat{X}}_{T+\Delta T}$ corresponds to the probability estimation of hit—the body part is on the path between ${\hat{x}}_{T}$ and each point of the red area. On the contrary, the blue area corresponds to no hit of the body. (Top) Top view. (Bottom) Side view. The silhouette’s reference frame (left) is placed to the torso.

To extend the model to three dimensions, we generalized 1D position and velocity to 3D vectors and the border of a body part is generalized to a 2D rectangle enclosed in 3D space—only the “collision plane”, not the depth of the body part is considered; see Fig 7. The details are in Section Extension to 3D space. We approximated the face by a rectangle with size [25*cm*, 25*cm*], and the torso by a rectangle with size [50*cm*, 50*cm*]. In contrast to the 1D scenario, now the object *can miss* the body part, which decreases the probability of hit. In all experiments, the object is moving along *x*_1_ axis to the center of the body part (see Fig 7). Therefore, if the position and velocity uncertainty in the vertical and horizontal axis are zero ($\sigma_{x,v}^{2,3}=0$), the probability estimation of hit is the same as in the 1D case, because missing the body part on the left/right or over/under it is excluded. This means that the variables related to the first dimension (e.g., $x_{T}^{1},v_{T}^{1},\sigma_{x}^{1}$) are equivalent to the variables of the 1D model (e.g., *x*_*T*_, *v*_*T*_, *σ*_*x*_). On the other hand, if the horizontal ($\sigma_{x,v}^{2}$) or vertical ($\sigma_{x,v}^{3}$) uncertainty increases, there is a corresponding stochastic estimate that the object may miss the body part and hence the estimation of probability of hit and of $y_{pred}^{*}$ goes down.

**Fig 8:**
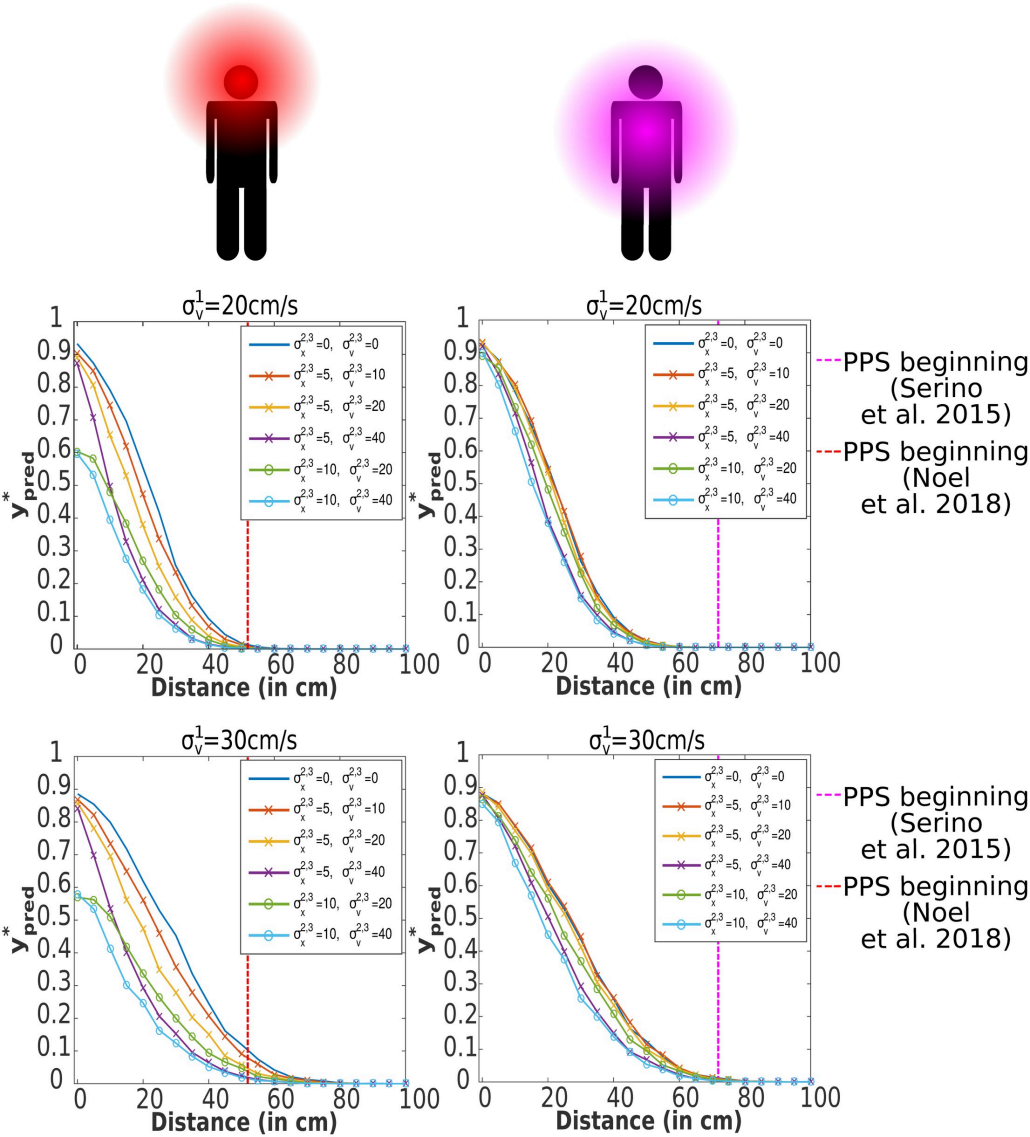
Modulation of PPS size by body part size in a 3D model (face and torso). For this experiment, 3D model was used (see Extension to 3D space). Dependency between distance of stimuli from body and the mean of 1000 impact predictions $y_{pred}^{*}$ for each distance and for PPS representation around the face (body part size [25cm, 25cm]) and trunk (body part size [50cm, 50cm]). The object is moving along the *x*_1_ axis ($x_{T}=\left[x_{T}^{1},x_{T}^{2}=0,x_{T}^{3}=0\right],v_{T}=\left[v_{T}^{1}=-25cm/s,v_{T}^{2}=0,v_{T}^{3}=0\right]$). Position and velocity estimation uncertainty for the first dimension are $\sigma_{x}^{1}=2.5cm$ and $\sigma_{v}^{1}=20cm/s$ for the first row, $\sigma_{v}^{1}=30cm/s$ for the second row. The uncertainties in the other two dimensions $\sigma_{x,v}^{2,3}$ (in cm or cm/s) are varied through the experiments. All other parameters are the baseline parameters from Table 1. The vertical dashed lines correspond to the estimations of the beginning of PPS from [14, 33]. See S4 Fig for a version with *FN* = *FP* = 1.

Experiments with this model are shown in Fig 8. In the first experiment, we used baseline parameters from the 1D case (see Table 1) and manipulated horizontal (axis *x*_2_) and vertical (axis *x*_3_) position and velocity estimation uncertainties (first row—$\sigma_{v}^{1}=20cm/s$—in Fig 8). For some settings of perceptual uncertainty, there is a difference in PPS size between the face and torso. However, for the torso, the beginning of PPS is still much smaller compared to the empirical value (72cm from [33]). In an effort to come close to the empirical values, we increased the velocity uncertainty in the first dimension from the baseline value to $\sigma_{v}^{1}=30cm/s$, leading to a general expansion of PPS (similarly to the experiment from Fig 6). For position and velocity uncertainties in the other dimensions, $\sigma_{x}^{2,3}=5cm,\sigma_{v}^{2,3}=40cm/s$ (purple curve in Fig 8), the beginning of face and torso PPS roughly fit empirical estimations (torso 72cm [33], face 52cm [14]). Thus, to fit empirical data, large horizontal and vertical velocity uncertainty $\sigma_{v}^{2,3}$ and small horizontal and vertical position uncertainty $\sigma_{x}^{2,3}$ are necessary. If the horizontal and vertical position uncertainty is further increased to $\sigma_{x}^{2,3}=10cm$, the maximal value of $y_{pred}^{*}$ is only 0.6 even for zero distance from the face, which would predict bigger reaction times in close proximity for the face than for the torso. We speculate that this is not plausible.

Two additional observations are in order. First, interestingly, our results suggest that horizontal and vertical uncertainty matters more for small body parts—something that can be empirically tested. Second, for low values of horizontal and vertical uncertainty, the 3D model for the torso has very similar PPS size and shape as the 1D case. Thus, a 3D model may often not be necessary.

### PPS shape modulated by prediction time step

**Fig 9:**
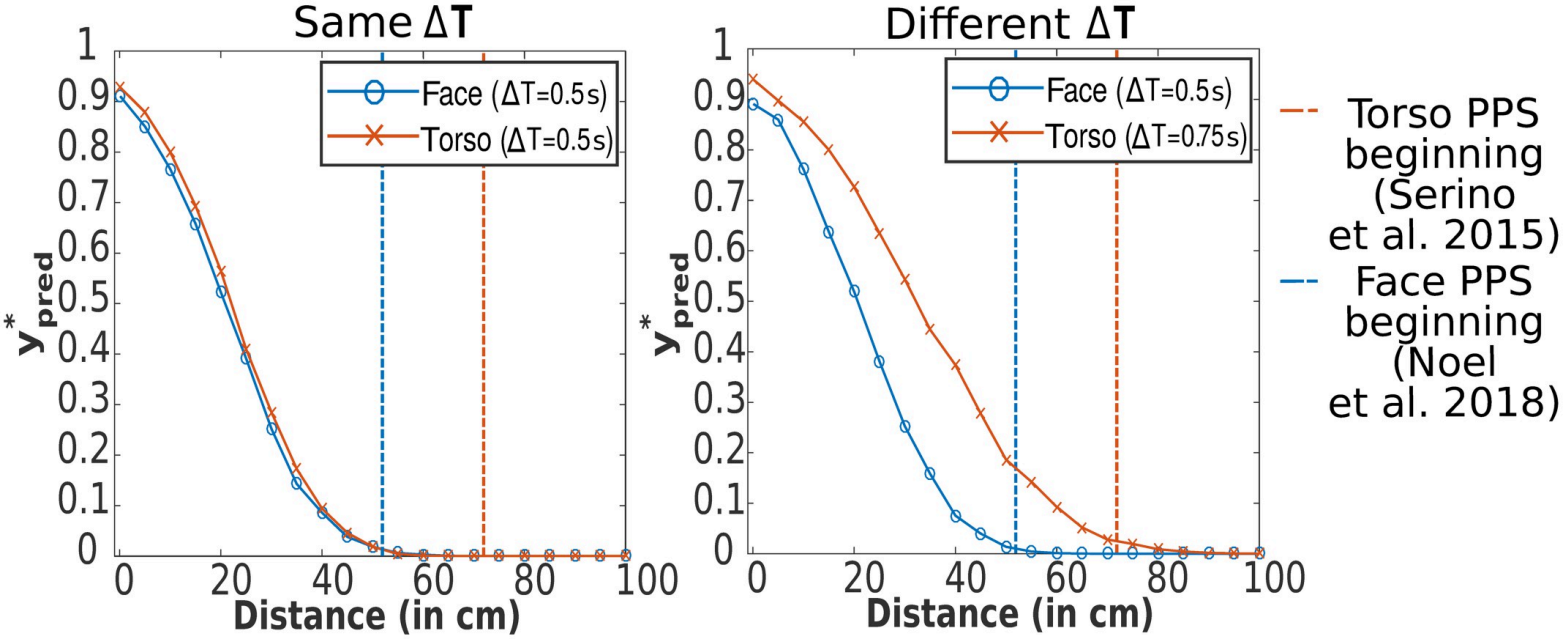
Modulation of PPS for face and torso in 3D model by prediction time step. Dependency between distance of stimuli from body and the mean of 1000 impact predictions $y_{pred}^{*}$ calculated by the 3D model (see Section Extension to 3D space) for each distance and for PPS representation around the face (body part size [25cm, 25cm]) and trunk (body part size [50cm, 50cm]). Baseline parameters (see Table 1) were used. Horizontal and vertical uncertainties were set to $\sigma_{x}^{2,3}=5cm,\sigma_{v}^{2,3}=5cm/s$. For a detailed experiment description see Fig 8. **(Left)** Prediction time step Δ*T* is same for both body parts (baseline value). The vertical and horizontal uncertainties are not large enough to cause different sizes for both body parts. **(Right)** The prediction time step is higher for the torso (Δ*T* = 0.75*s*) than for the face. In this setting, the PPS beginnings of both body parts fit roughly the empirical estimations. The vertical dashed lines correspond to the PPS beginning estimations from [14, 33]. See S5 Fig for a version with *FN* = *FP* = 1.

An alternative parameter that could potentially influence the different extent of PPS is the prediction time step parameter Δ*T* (in our model it was fixed to 0.5s). It may be interpreted as the time the agent needs to perform a defensive action that will protect the body part threatened by the impending collision. The effects of Δ*T* ∈ {0.25, 0.5, 1}*s* on the 1D model are shown in S7 Fig (for the corresponding figure with *FN* = *FP* = 1 see S8 Fig). Depending on the body part and the action, the “time constant” may differ. For example, blinking to protect the eyes will be faster than squatting to protect the whole torso. To explore this hypothesis, we performed an experiment with Δ*T* = 0.5*s* for the face and Δ*T* = 0.75*s* for the torso on the 3D model—see Fig 9. It is apparent that the Δ*T* parameter is very effective in shifting the PPS boundary.

## Discussion

Understanding how observers avoid collision with approaching environmental objects potentially harming their bodies is of paramount importance in furthering our understanding of self-environment interactions. It has long been postulated that neurons encoding for our PPS may play a critical role in this computation [4, 9, 14, 45, 46]. Yet, there has been no formal, normative demonstration. In turn, the major contribution of the current work is the derivation of a Bayes optimal model of impact prediction that consists of impact probability estimation and a cost function simulating the utility/penalty for the agent incurred by the impending collision. Supporting the hypothesis that PPS encodes the prediction of future contact, in a value-dependent manner, the normative model of impact prediction can recapitulate several of the defining characteristics of PPS: (i) a graded delineation of near and far space [37], a preference for (ii) approaching [6, 29, 30] and (iii) rapidly moving [10, 14] stimuli, (v) a scaling of the “boundary” differentiating near and far space as a function of the valence attributed to the approaching object [31, 32], and finally (v) differing sizes for different body parts [33]. The model also makes a set of concrete and testable hypotheses for future work. For instance, the fact that stimuli velocity ought to impact PPS delineation differently for looming and receding trajectories (see Fig 6), the fact that perceptual uncertainty ought to have an impact on PPS size and boundary shape (see Fig 4A) and that perceptual uncertainty in orthogonal directions to the looming object impacts more the characteristics of PPS for smaller rather than larger body parts (Fig 8), and finally, the fact that “sensory” and “cognitive” effects ought to shape PPS encoding differently (compare Fig 4A and 4B).

Interestingly, the derivation highlights two major factors (beyond the environmental, such as the position and velocity of incoming stimuli, as well as the size of body parts) that may largely determine the shape and size of PPS. First, aspects related to the loss function—the value attributed to false positive vs. false negative detection of contact (see [37] for an opinion piece proposing a value-based theory of PPS). This loss function is likely modulated by social, emotional, motor, attentional, and even reflex-like computations that ascribe a value to, or a danger associated with, objects and events in the environment (see [4, 5] for further discussion). Second, aspects related to the precision with which an observer may estimate the position and velocity of the approaching object and self-position. Conveniently, these two factors affect the overall size of PPS (e.g., the central point of a sigmoidal function differentiating between the near and far space) and its gradient (e.g., the slope of the sigmoid) differently. While the value-based computation may modulate the overall size of PPS, it only minimally affects the gradient between near and far space. On the other hand, if an enlargement of PPS is due to changes in low-level sensory uncertainty, by necessity this has to be accompanied by a flattening of the curve differentiating between the near and far space. The differing effect engendered by changes in the loss function vs. computing the probability of contact should allow researchers to attribute their empirical effects to one or the other component of the normative impact prediction model. In S6 Fig, we provide 3D plots illustrating the effects of velocity uncertainty (*σ*_*v*_), false positive cost (*FN*), and prediction time step (Δ*T*) on the slope of the PPS boundary and its size.

Manipulations intended to affect the loss function are commonplace in PPS research [31, 32]—even if not necessarily conceived as such. For instance, researchers have presented observers with sights or sounds of objects approaching with either a positive, neutral, or negative valence. Examining this literature under the current framework suggests that while ecological stimuli may in fact affect solely the loss function (i.e., changes in the false negative parameter, modulating only PPS size but not the shape of the boundary), artificial stimuli may affect both value-based computation, as well as the precision of sensory representations (see PPS encoding and object value).

More notoriously, the current framework points to a large empirical void. That is, while a critical element of the current model, there is a lack of studies examining how sensory uncertainty—by e.g., varying size, contrast, adding observation noise, or making the approach trajectory variable—may affect PPS (but see Huijsmans et al. [7] for a recent exception). The normative model of impact prediction would hypothesize that more uncertain stimuli should lead to a larger PPS, depending on how the size of PPS is operationalized—cf. Section A graded PPS “boundary”—Effect of sensory uncertainty and cost of false negative prediction. To the best of our knowledge, this has not been explicitly tested. However, Schlack et al. [47], did record from single cells in the ventral intra-parietal area—an area known to house PPS neurons (see e.g., [6])—while presenting auditory or visual stimuli (the former being more imprecisely localized in space, [48]). The authors reported larger auditory than visual receptive fields in this area, suggesting that audio-tactile PPS may be wider than visuo-tactile PPS, as the normative model of impact prediction would conjecture.

On the modeling front, PPS is commonly associated with not only defensive [6], but also with approaching behaviors [34]. Thus, in the future we may develop a full choice model, where an agent does not only predict if impact will occur or not, but could also take either avoiding or approaching actions. In this line, Roncone et al. [19] made a robot move toward or away from objects by connecting artificial “PPS neurons” to a controller. In our case, now equipped with a normative model of impact prediction, we could trigger actions based on a specific value of $y_{pred}^{*}$. Two aspects of the current work are worth highlighting in this action-oriented setting. First, here we either used a loss function where *FN* > *FP* or an unbiased one (*FN* = *FP*). However, this need not always be the case. In particular when approaching objects, the cost associated with “miss” may be higher than that associated with a “false positive”. Namely, a striking difference between “PPS for defensive behavior” and “PPS for action” may be that in the former *FN* > *FP* while in the latter *FN* < *FP*. Second, we ought to highlight that in order to qualitatively match empirical estimates of PPS sizes across different body parts, varying the Δ*T* parameter was more effective than the *FN*/*FP* ratio. For defensive PPS, this parameter may be mainly motivated by the time needed to trigger and execute a protective action. This may differ for body parts—protecting the torso by moving it requires whole-body action, while hand or head could be protected relatively more easily—or even for the same body parts depending on context, such as the character of a potential threat. For example, protecting the eyes against flying sand by blinking is more rapid than a squatting action or moving the arms in front of the face when the threat is different. Similarly, in invasive single cell recordings a striking feature of PPS neurons is their vast heterogeneity in receptive field sizes. Our current results suggest that perhaps akin to what is observed in other spatial codes (e.g., place or grid cells) this heterogeneity bears from different intrinsic time-scales of each neuron.

It is also worth noting that the our model predicts complete curves relating impact prediction and distance of the object from the body. It generates empirical predictions about how different parameters such as perceptual uncertainty or object valence modify this curve—by offsets along the distance axis, change in its slope, or their combination. To test the model predictions in real experiments, complete distance-dependent curves are desired, as opposed to simplifications defining PPS boundaries as either the farthest distance with an effect on a measured variable or as a midpoint of a fitted sigmoidal curve. Reducing the response curve to a single distance may blur the impact of the different factors.

In conclusion, we derived a normative model of impact prediction, and demonstrated that this model accounted for a number of characteristics of PPS. Further, this exercise highlighted that beyond characteristics of the environment itself, the two main factors influencing PPS size and shape are (i) the ability to represent the external environment precisely, and (ii) the value attributed to false positive and negatives. Conveniently, these factors express differently (either affecting both size and shape of PPS, or solely size), and thus researchers ought to be able to attribute their effects to one or the other. Further, our formal approach has highlighted aspects of empirical work that are still missing, most notoriously the ability to index biases and variance in PPS on the individual subject level. We hope novel methods to index PPS are developed (e.g., estimation tasks), which will allow for further joint theory—experiment examination of impact prediction and PPS encoding.

## Materials and methods

### Derivation of the normative impact prediction model

In line with the probabilistic (e.g., [21]) framework to perception, we propose an estimation procedure of computing the probability of future impact on the body (see Fig 1 for a schema with an example). Following the estimation procedure, Bayesian Decision Theory (e.g., [25]) is employed for impact prediction calculation.

An external object is moving on a straight line toward or away from the body. At time *T*, a stimulus has position *x*_*T*_ ∈ ℝ (distance from the body) and moves with velocity *v*_*T*_ ∈ ℝ (negative values for a looming object). We followed [21] (among others) and supposed that sensory estimations of the position ${\hat{x}}_{T}$ and velocity ${\hat{v}}_{T}$ are corrupted by Gaussian noise with variances $\sigma_{x}^{2}$ and $\sigma_{v}^{2}$, respectively. To simulate the effect of noise, ${\hat{x}}_{T}$ and ${\hat{v}}_{T}$ were obtained as samples from normal distributions *N*(*μ* = *x*_*T*_, *σ* = *σ*_*x*_) and *N*(*μ* = *v*_*T*_, *σ* = *σ*_*v*_). If the object position sample is within the body (${\hat{x}}_{T}<0$), it is set to ${\hat{x}}_{T}=0.1cm$—immediately in front of the body. Notice that the higher values (e.g., auditory localization as opposed to visual localization) of the standard deviations *σ*_*x*_, *σ*_*v*_ are related to less precise estimations.

The brain does not only encode point estimates, but also their uncertainties [21, 23, 24, 49]. Hence, we did not use only the point estimates ${\hat{x}}_{T},{\hat{v}}_{T}$ of the position and velocity, but also included the uncertainty caused by the observation noise—the estimates of the position and velocity are encoded as normal distributions $N(\mu ={\hat{x}}_{T},\sigma =\sigma_{x})$, $N(\mu ={\hat{v}}_{T},\sigma =\sigma_{v})$, respectively.

Next, we compute an estimate of object displacement during Δ*T*. The displacement is encoded as $N(\mu =\Delta T\cdot {\hat{v}}_{T},\sigma =\Delta T\cdot \sigma_{v})$. Note that this estimation, based on the equation *displacement* = Δ*T* ⋅ *velocity*, is precise only if the velocity does not change during Δ*T* (as assumed in the current simulations and in all empirical studies of PPS with approaching objects).

Given the estimate of the initial position and displacement of the object, we can estimate its future position, ${\hat{X}}_{T+\Delta T}$. This position is calculated as *position*_*T*+Δ*T*_ = *position*_*T*_ + *displacement*. In case of Gaussian random variables, this means ${\hat{X}}_{T+\Delta T}∼N\left(\mu ={\hat{x}}_{T}+\Delta T\cdot {\hat{v}}_{T},\sigma =\sqrt{\sigma_{x}^{2}+{\left(\Delta T\cdot \sigma_{v}\right)}^{2}}\right)$. Notice that the calculation of the overall estimation uncertainty $\sigma =\sqrt{\sigma_{x}^{2}+{\left(\Delta T\cdot \sigma_{v}\right)}^{2}}$ shows that manipulations of *σ*_*v*_ (used in some simulations) is interchangeable with manipulations of *σ*_*x*_ (only Δ*T* has to be taken into account). Therefore, the qualitative effects engendered by manipulating velocity uncertainty *σ*_*v*_ in the main text can be generalized to position uncertainty *σ*_*x*_. The model restricts mean of position estimation to only the space in front of the body.

We can estimate the probability of impact, $P(Y|\left({\hat{x}}_{T},\sigma_{x}\right),\left({\hat{v}}_{T},\sigma_{v}\right))$, where *Y* ∈ {0, 1} represents whether the object hits the body (*y* = 1) or not (*y* = 0). As the prediction is calculated before the object hits (or not) the body, the actual future impact value *y* is not known during the calculation. Therefore, the calculation takes into account the estimated probability $P(y|\left({\hat{x}}_{T},\sigma_{x}\right),\left({\hat{v}}_{T},\sigma_{v}\right))$ for both possible values of *y*. It is estimated as $P\left(y=1|\left({\hat{x}}_{T},\sigma_{x}\right),\left({\hat{v}}_{T},\sigma_{v}\right)\right)=P\left({\hat{X}}_{T+\Delta T}\leq 0\right)$. That is, this is the estimation that the object will be on the surface of the body or farther in space (see Fig 1) at time *T* + Δ*T*. Namely, contact of the object with the body can occur at any time between time *T* and *T* + Δ*T*. The probability estimation that the body will not be hit is $P\left(y=0|\left({\hat{x}}_{T},\sigma_{x}\right),\left({\hat{v}}_{T},\sigma_{v}\right)\right)=1-P\left(y=1|\left({\hat{x}}_{T},\sigma_{x}\right),\left({\hat{v}}_{T},\sigma_{v}\right)\right)$. Given the above, according to Bayesian Decision Theory [25, 26], the optimal decision—in our case the impact prediction $y_{pred}^{*}\in \left[0,1\right]$—is calculated as

$$\begin{array}{c} y_{pred}^{*}=\text{arg}\underset{y_{pred}\in \left[0,1\right]}{\text{min}}L\left(\left({\hat{x}}_{T},\sigma_{x}\right),\left({\hat{v}}_{T},\sigma_{v}\right),y_{pred}\right) \end{array}$$ (5)

where $L(\left({\hat{x}}_{T},\sigma_{x}\right),\left({\hat{v}}_{T},\sigma_{v}\right),y_{pred})$ can be further expanded in the following manner by using a loss function definition

$$\begin{array}{c} L\left(\left({\hat{x}}_{T},\sigma_{x}\right),\left({\hat{v}}_{T},\sigma_{v}\right),y_{pred}\right)=P\left(y=1|\left({\hat{x}}_{T},\sigma_{x}\right),\left({\hat{v}}_{T},\sigma_{v}\right)\right)\cdot loss\left(y=1,y_{pred}\right)+\\ P\left(y=0|\left({\hat{x}}_{T},\sigma_{x}\right),{\hat{V}}_{T}\right)\cdot loss\left(y=0,y_{pred}\right)=\\ P\left(y=1|\left({\hat{x}}_{T},\sigma_{x}\right),\left({\hat{v}}_{T},\sigma_{v}\right)\right)\cdot loss\left(y=1,y_{pred}\right)+\\ \left(1-P\left(y=1|\left({\hat{x}}_{T},\sigma_{x}\right),{\hat{V}}_{T}\right)\right)\cdot loss\left(y=0,y_{pred}\right)=\\ P\left(y=1|\left({\hat{x}}_{T},\sigma_{x}\right),\left({\hat{v}}_{T},\sigma_{v}\right)\right)\left(FP\;\text{max}{\left(0,y_{pred}-1\right)}^{2}+FN\;\text{max}{\left(0,1-y_{pred}\right)}^{2}\right)+\\ \left(1-P\left(y=1|\left({\hat{x}}_{T},\sigma_{x}\right),\left({\hat{v}}_{T},\sigma_{v}\right)\right)\right)\left(FP\;\text{max}{\left(0,y_{pred}-0\right)}^{2}+FN\;\text{max}{\left(0,0-y_{pred}\right)}^{2}\right)=\\ P\left(y=1|\left({\hat{x}}_{T},\sigma_{x}\right),\left({\hat{v}}_{T},\sigma_{v}\right)\right)FN{\left(1-y_{pred}\right)}^{2}+\left(1-P\left(y=1|\left({\hat{x}}_{T},\sigma_{x}\right),\left({\hat{v}}_{T},\sigma_{v}\right)\right)\right)FPy_{pred}^{2} \end{array}$$ (6)

A prediction, *y*_*pred*_ = 1 corresponds to hit prediction, is evaluated according to a function *loss*: *Y* × *Y*_*pred*_ → [0, ∞) which determines the cost incurred (or penalty) when the predicted value *y*_*pred*_ does not correspond to the future tactile impact value *y*. In other words, the loss function reflects the difference between the predicted tactile activation and the actual future tactile activation *y* at time *T* + Δ*T*. The loss function is expressed as

$$\begin{array}{c} loss\left(y,y_{pred}\right)=FP\;\text{max}{\left(0,y_{pred}-y\right)}^{r}+FN\;\text{max}{\left(0,y-y_{pred}\right)}^{r} \end{array}$$ (7)

where *FP, FN* ∈ [0, ∞] are respectively the false positive and false negative factors, max(0, *x*) is a function which outputs *x* for *x* ≥ 0 and 0 for *x* < 0. The parameter *r* ∈ (0, ∞) shapes the loss function. Throughout the simulations, we maintained it fixed to *r* = 2. If the prediction matches the actual impact value, the loss will be 0. Instead, if *y*_*pred*_ > *y*, then the loss function (7) is reduced to *loss*(*y*, *y*_*pred*_) = *FP*(*y*_*pred*_ − *y*)^2^ and the maximal value is reached when tactile contact is predicted (*y*_*pred*_ = 1) but does not happen (*y* = 0). Lastly, if *y*_*pred*_ < *y*, then the loss function (7) is equal to *loss*(*y*, *y*_*pred*_) = *FN*(*y* − *y*_*pred*_)^2^ and the loss is maximal when contact occurs (*y* = 1) without a prediction of this happening (*y*_*pred*_ = 0). We suggest that the loss during *FN* cases is higher than during *FP* cases because objects making contact with the body without any prediction—thus no defensive action—may be more harmful than making predictions of contact that do not in fact occur.

Note that the prediction is optimal in relation to the estimated probability $P(y|\left({\hat{x}}_{T},\sigma_{x}\right),\left({\hat{v}}_{T},\sigma_{v}\right))$ of (no) impact given the object position and velocity estimations. Because these sensory estimations are stochastic (point estimations ${\hat{x}}_{T},{\hat{v}}_{T}$ of *x*_*T*_, *v*_*T*_ are corrupted by Gaussian noise), there are multiple predictions $y_{pred}^{*}$ for one position *x*_*T*_ and velocity *v*_*T*_ and all of them are optimal in relation to the object position and velocity estimations $N\left(\mu ={\hat{x}}_{T},\sigma_{x}\right),N\left(\mu ={\hat{v}}_{T},\sigma_{v}\right)$ of *x*_*T*_ and *v*_*T*_, respectively.

### Extension to 3D space

The model proposed above is one-dimensional. We extended this model to three dimensions. It means that both position and velocity are represented by 3-dimensional vectors $x_{T}=\left[x_{T}^{1},x_{T}^{2},x_{T}^{3}\right]$ and $v_{T}=\left[v_{T}^{1},v_{T}^{2},v_{T}^{3}\right]$. In our model, the movement in each dimension is treated equivalently to the movement in the 1D model and independently on other dimensions (see the selected reference frame in Fig 7). Therefore, position and velocity point estimates ${\hat{x}}_{T}=\left[{\hat{x}}_{T}^{1},{\hat{x}}_{T}^{2},{\hat{x}}_{T}^{3}\right],{\hat{v}}_{T}=\left[{\hat{v}}_{T}^{1},{\hat{v}}_{T}^{2},{\hat{v}}_{T}^{3}\right]$ are sampled independently in individual dimensions depending on the position and velocity uncertainties $\sigma_{x}=\left[\sigma_{x}^{1},\sigma_{x}^{2},\sigma_{x}^{3}\right]$, $\sigma_{v}=\left[\sigma_{v}^{1},\sigma_{v}^{2},\sigma_{v}^{3}\right]$.

The three-dimensional generalization ${\hat{X}}_{T+\Delta T}$ of the one-dimensional future position estimation ${\hat{X}}_{T+\Delta T}∼N\left(\mu ,\sigma \right)$ is distributed as a multivariate normal distribution with a diagonal covariance matrix (see Fig 7)

$$\begin{array}{c} {\hat{X}}_{T+\Delta T}∼N(\begin{array}{c} \mu_{1}={\hat{x}}_{T}^{1}+\Delta T\cdot {\hat{v}}_{T}^{1},\sigma_{1}=\sqrt{{\left(\sigma_{x}^{1}\right)}^{2}+{\left(\Delta T\cdot \sigma_{v}^{1}\right)}^{2}}\\ \mu_{2}={\hat{x}}_{T}^{2}+\Delta T\cdot {\hat{v}}_{T}^{2},\sigma_{2}=\sqrt{{\left(\sigma_{x}^{2}\right)}^{2}+{\left(\Delta T\cdot \sigma_{v}^{2}\right)}^{2}}\\ \mu_{3}={\hat{x}}_{T}^{3}+\Delta T\cdot {\hat{v}}_{T}^{3},\sigma_{3}=\sqrt{{\left(\sigma_{x}^{3}\right)}^{2}+{\left(\Delta T\cdot \sigma_{v}^{3}\right)}^{2}} \end{array}) \end{array}$$ (8)

The body part is represented as a rectangle with size [2 ⋅ *r*_1_, 2 ⋅ *r*_2_] (see Fig 7). The probability of a hit is estimated as

$$\begin{array}{c} P\left(y=1|\left({\hat{x}}_{T},\sigma_{x}\right),\left({\hat{v}}_{T},\sigma_{v}\right)\right)=\int_{-\infty }^{0}\int_{b_{1}x_{1}-r_{1}}^{a_{1}x_{1}+r_{1}}\int_{b_{2}x_{1}-r_{2}}^{a_{2}x_{1}+r_{2}}f_{{\hat{X}}_{T+\Delta T}}\left(x_{1},x_{2},x_{3}\right)\;dx_{1}\;dx_{2}\;dx_{3},=\\ \int_{-\infty }^{0}\int_{b_{1}x_{1}-r_{1}}^{a_{1}x_{1}+r_{1}}\int_{b_{2}x_{1}-r_{2}}^{a_{2}x_{1}+r_{2}}f_{N(\mu_{1},\sigma_{1})}\left(x_{1}\right)\cdot f_{N(\mu_{2},\sigma_{2})}\left(x_{2}\right)\cdot f_{N(\mu_{3},\sigma_{3})}\left(x_{3}\right)\;dx_{1}\;dx_{2}\;dx_{3}, \end{array}$$ (9)

where *a*_1_, *a*_2_, *b*_1_, *b*_2_ are the parameters of the integration boundaries (see Fig 7 for details) and *f* represents the probability density function. The probability of no hit can be calculated as $P\left(y=0|\left({\hat{x}}_{T},\sigma_{x}\right),\left({\hat{v}}_{T},\sigma_{v}\right)\right)=1-P\left(y=1|\left({\hat{x}}_{T},\sigma_{x}\right),\left({\hat{v}}_{T},\sigma_{v}\right)\right)$.

In our simulations, to speed up the probability calculation determined by the integral from Eq 9 and avoid problems (for example, zero horizontal and vertical uncertainties), we used numerical calculation. We generated 10000 samples for each future position estimation. The probability was estimated as a rate of samples within the “hit” area to all samples (see the code).

### Simulation details

In the simulations, we mimicked the setup of empirical reports. An object was approaching or receding from the body with constant velocity *v*_*T*_. In one experimental trial, for each distance *x*_*T*_ (e.g., 0, 5, 10, …, *x*_*max*_ cm) from the body, an impact prediction $y_{pred}^{*}$ was calculated. Notice that the choice of the *x*_*max*_ (beginning of the trajectory, in case of looming stimuli) did not affect the computed values of $y_{pred}^{*}$, because the predicted values depend only on the actual position and velocity (which is constant) and not on the previous trajectory.

Because the predictions $y_{pred}^{*}$ differ from trial to trial—similarly to measures in experiments with human observers—multiple trials for every experimental condition were performed. To summarize multiple predicted values $y_{pred}^{*}$ for each distance *x*_*T*_, means of $y_{pred}^{*}$ and 25th/75th percentiles for each distance *x*_*T*_ were calculated. In simulations, the expected loss (Eq (6)) is calculated for *y*_*pred*_ ∈ {0, 0.05, 0.1, …, 1} (except the experiment in Fig 2 where the granularity is 0.001) and the one with the smallest loss is then selected as the optimal value $y_{pred}^{*}$. A detailed example of $y_{pred}^{*}$ calculation with all details is in S1 and S2 Files (interactive version).

## Supporting information

S1 File A detailed example of an impact prediction calculation—Interactive version. (PDF)

S2 File A detailed example of an impact prediction calculation. For a more interactive version see S1 File.
(PDF)

S1 Fig A version of Fig 2 with *FN* = *FP* = 1. (EPS)

S2 Fig A version of Fig 6 with *FN* = *FP* = 1. (EPS)

S3 Fig A version of Fig 5 with *FN* = *FP* = 1. The vertical dashed lines correspond to the PPS beginning estimations from [14].
(EPS)

S4 Fig A version of Fig 8 with *FN* = *FP* = 1. The vertical dashed lines correspond to the PPS beginning estimations from [14, 33].
(EPS)

S5 Fig A version of Fig 9 with *FN* = *FP* = 1. The vertical dashed lines correspond to the PPS beginning estimations from [14, 33].
(EPS)

S6 FigSize of PPS and slope of its boundary is modulated by FN, Δ*T* and *σ*_*v*_.Beginning of PPS is determined as the farthest distance *x*_*T*_ for which the mean value of 1000 $y_{pred}^{*}$ samples overcomes 0.01. For slope calculation, mean values of 1000 $y_{pred}^{*}$ samples for each distance *x*_*T*_ were used. The slope was calculated around the central value (between min and max) of the curve. Technically, the slope was negative—the values were decreasing from left to right—in all cases. To better visualize the slope, we plotted absolute values of the slope. Except for *σ*_*v*_, Δ*T*, *FN* and *σ*_*x*_ = 0*cm*, the baseline parameters (see Table 1) were used. See the code for details.(EPS)

S7 FigEffect of timestep Δ*T* size on PPS.Dependency between the mean of 1000 predicted tactile activations $y_{pred}^{*}$ (for each distance) and distance *x*_*T*_ (in centimeters) of the stimuli from the body. The symbol “+” indicates 25th and 75th percentiles which are calculated from 1000 predicted values $y_{pred}^{*}$ for each distance. PPS size expands with increasing size of timestep Δ*T* (in seconds). Sharpness of the PPS boundary is decreasing with increasing size of timestep Δ*T*. Except for Δ*T*, baseline parameters are used (Table 1).(EPS)

S8 Fig A version of S7 Fig with *FN* = *FP* = 1. (EPS)
