# Supplementary material for: A normative model of peripersonal space encoding as performing impact prediction
Source: PLoS Comput Biol. 2022 Sep 14;18(9):e1010464. doi: 10.1371/journal.pcbi.1010464 (PMC9512250; doi:10.1371/journal.pcbi.1010464)
Supplement: S1 File — (PDF) [file pcbi.1010464.s001.pdf]

Example of impact prediction calculation

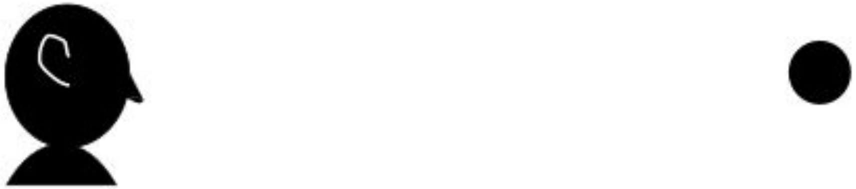

An object is looming to the body.

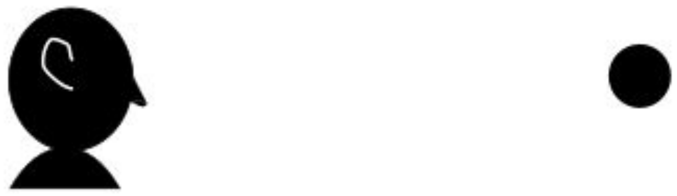

An object is looming to the body.

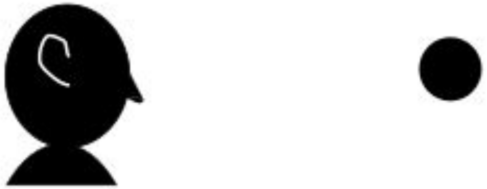

An object is looming to the body.

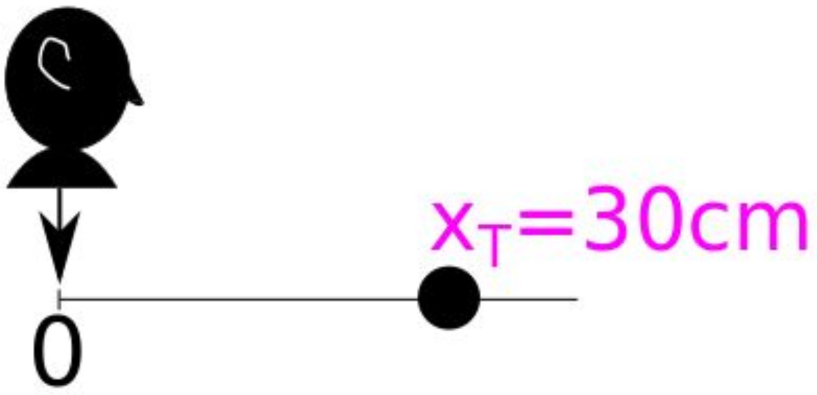

At time T, it is 30 cm from the body.

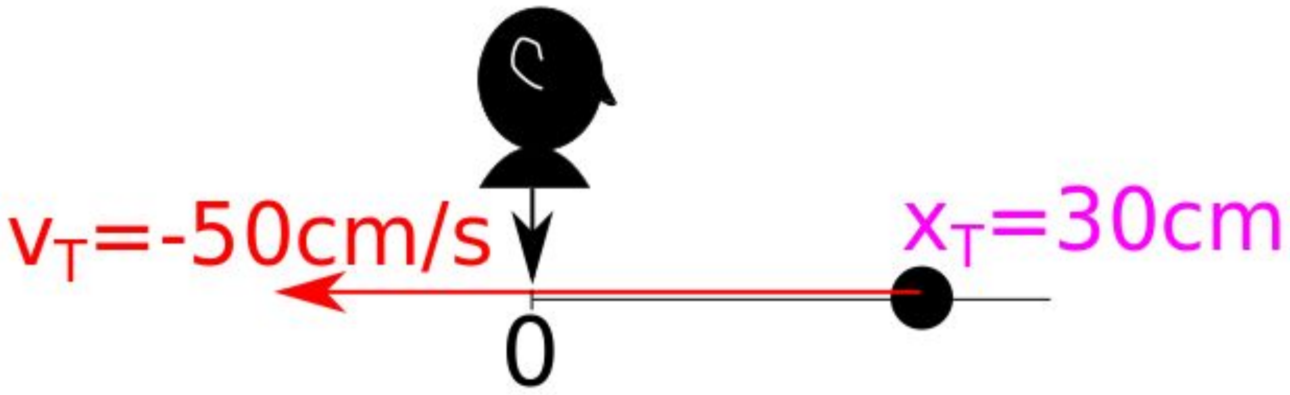

At time T, it is 30 cm from the body and its velocity is -50 cm/s.

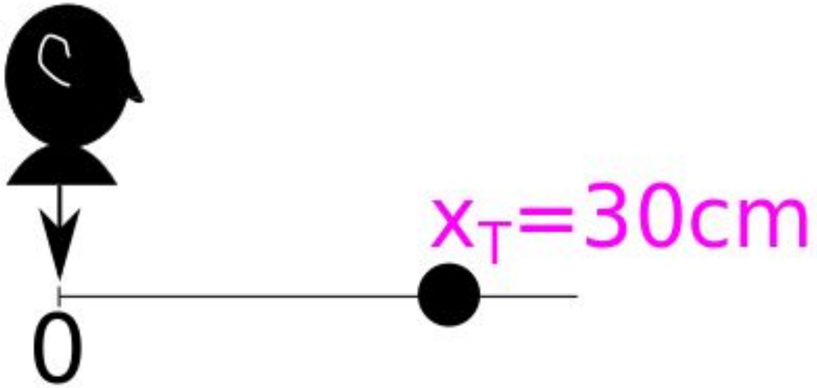

Let's start with the position estimation.

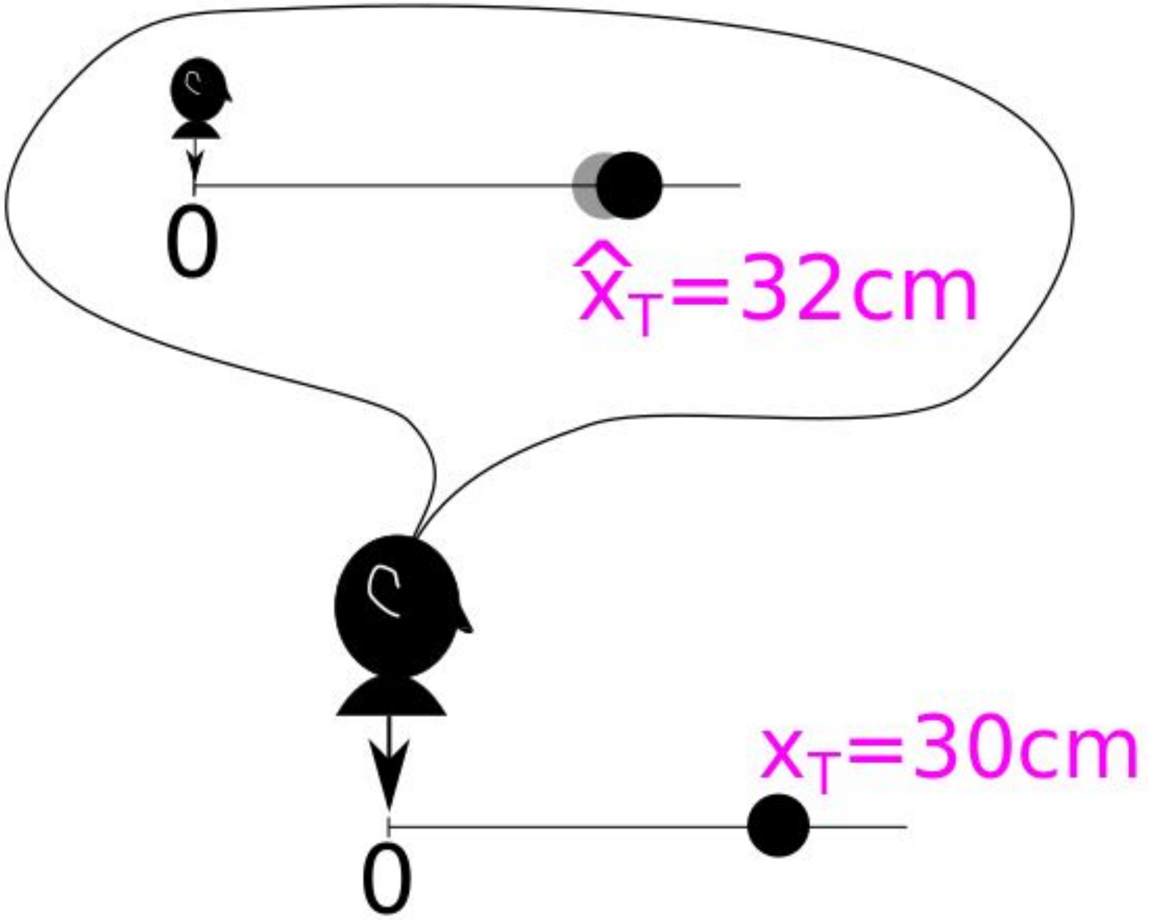

Assuming that the perception is noisy (Gaussian noise with standard deviation σ_x=4cm), the position is estimated as 32cm (sample from the normal distribution).

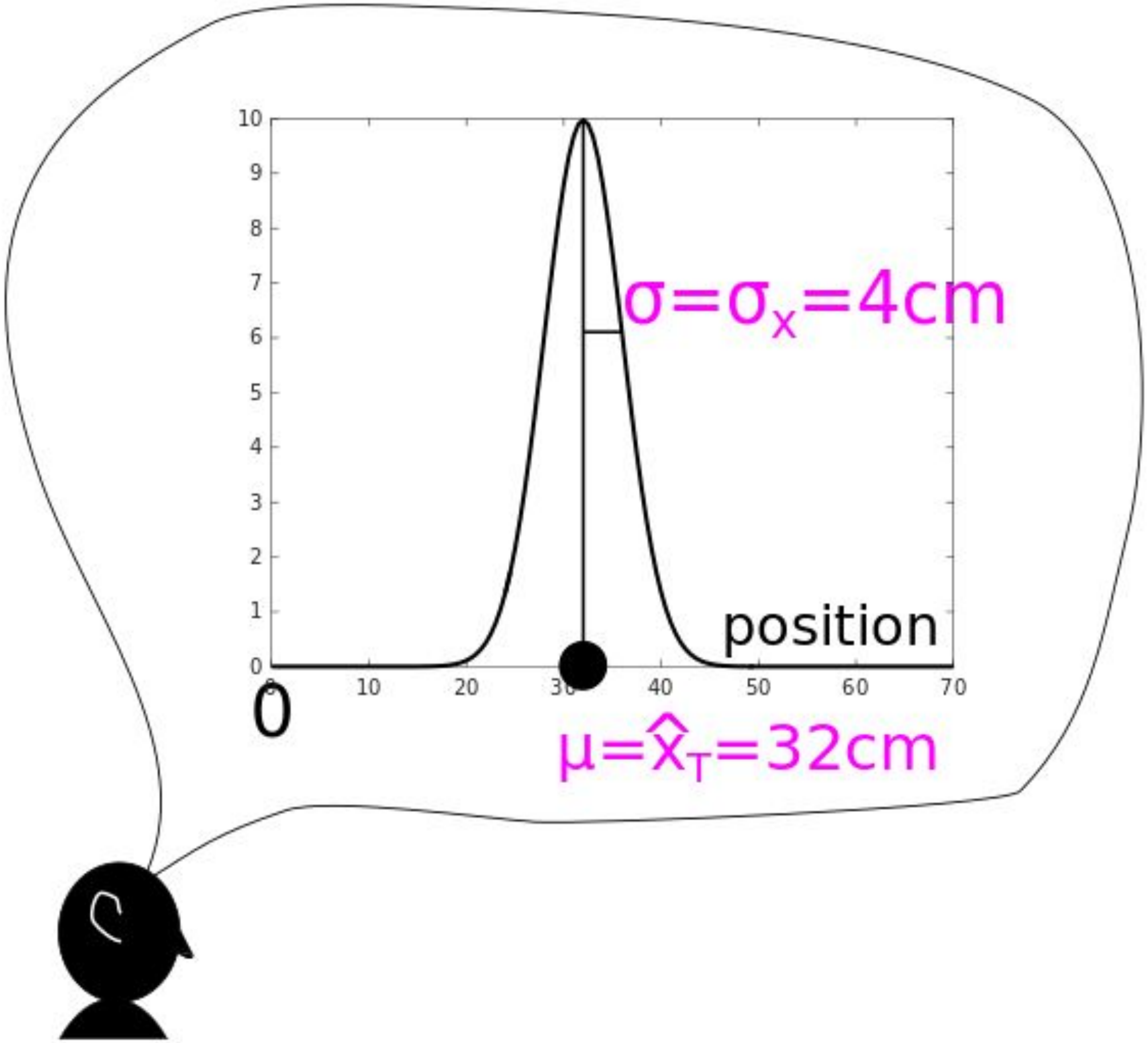

To include the estimate uncertainty, the estimate is encoded as a normal distribution.

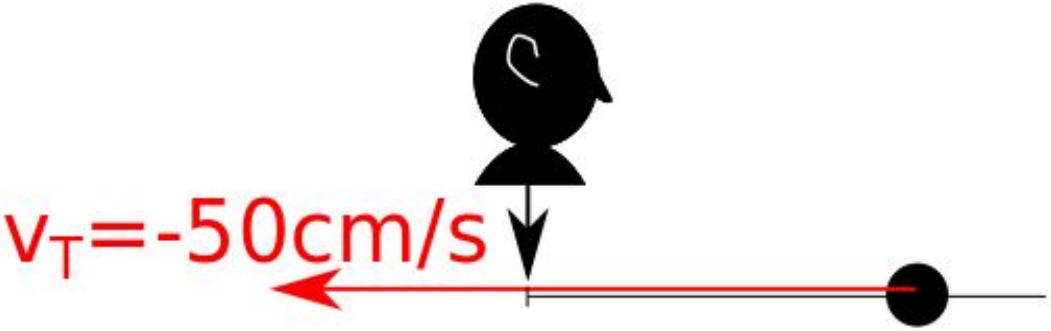

Let us add velocity.

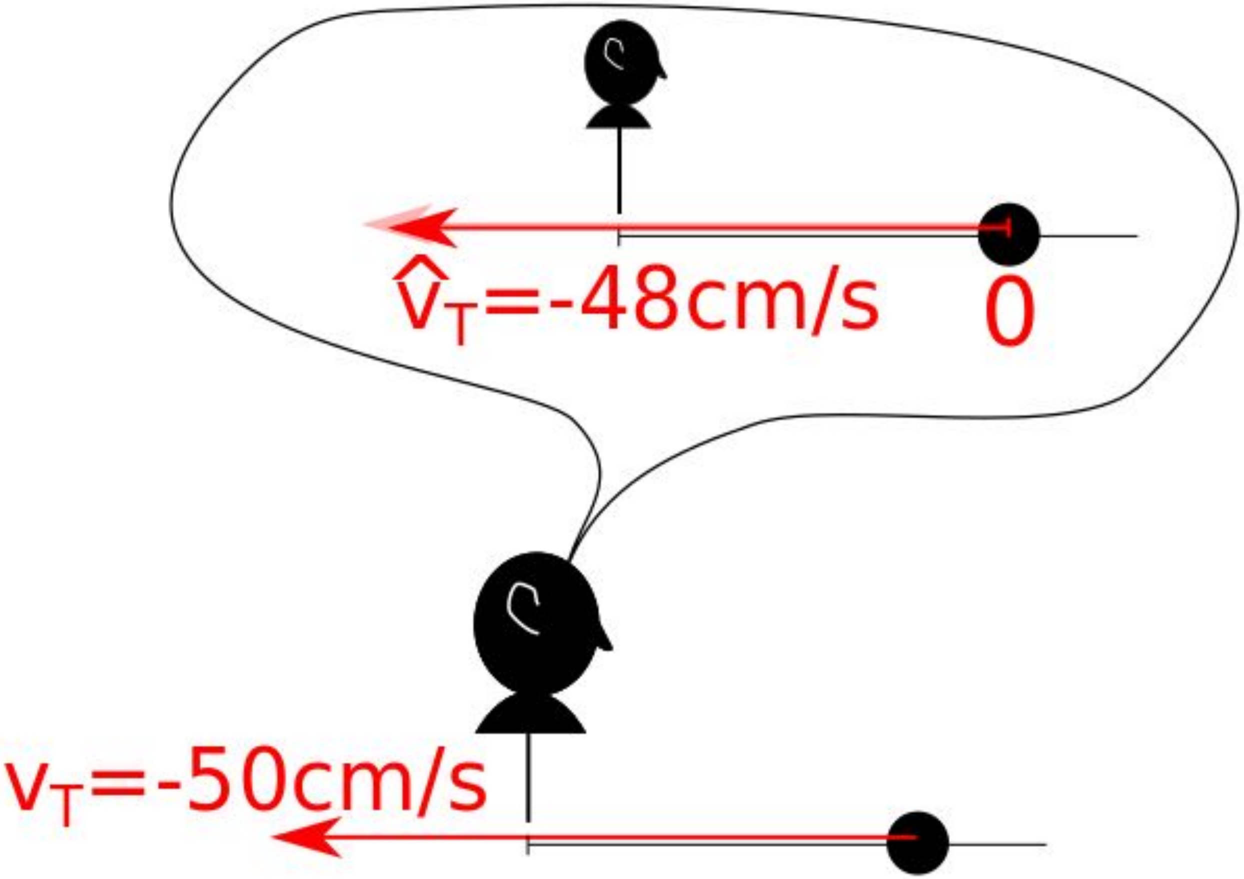

Velocity estimation is also noisy
(Gaussian noise with standard deviation σ_v=5cm/s).

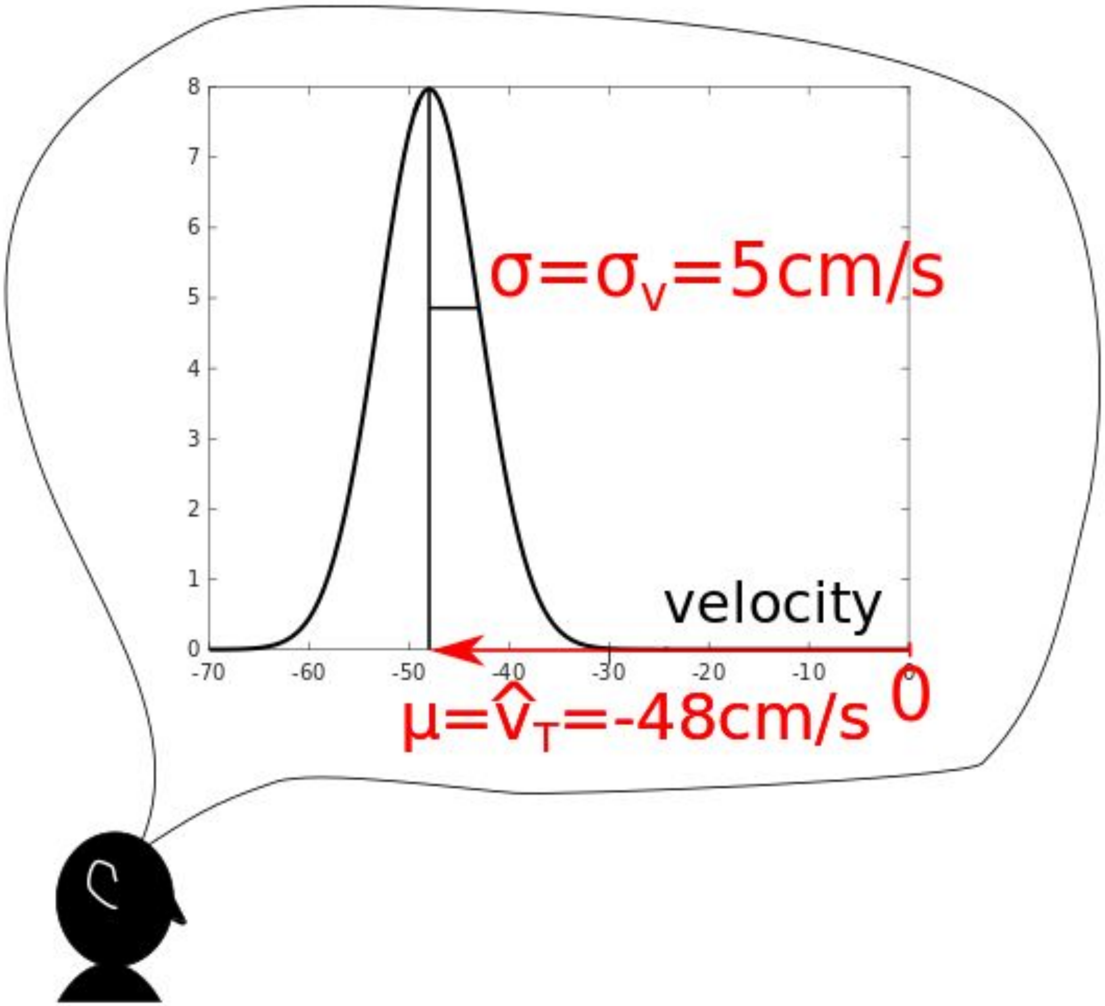

To include estimation uncertainty, velocity estimation is also encoded as normal distribution.

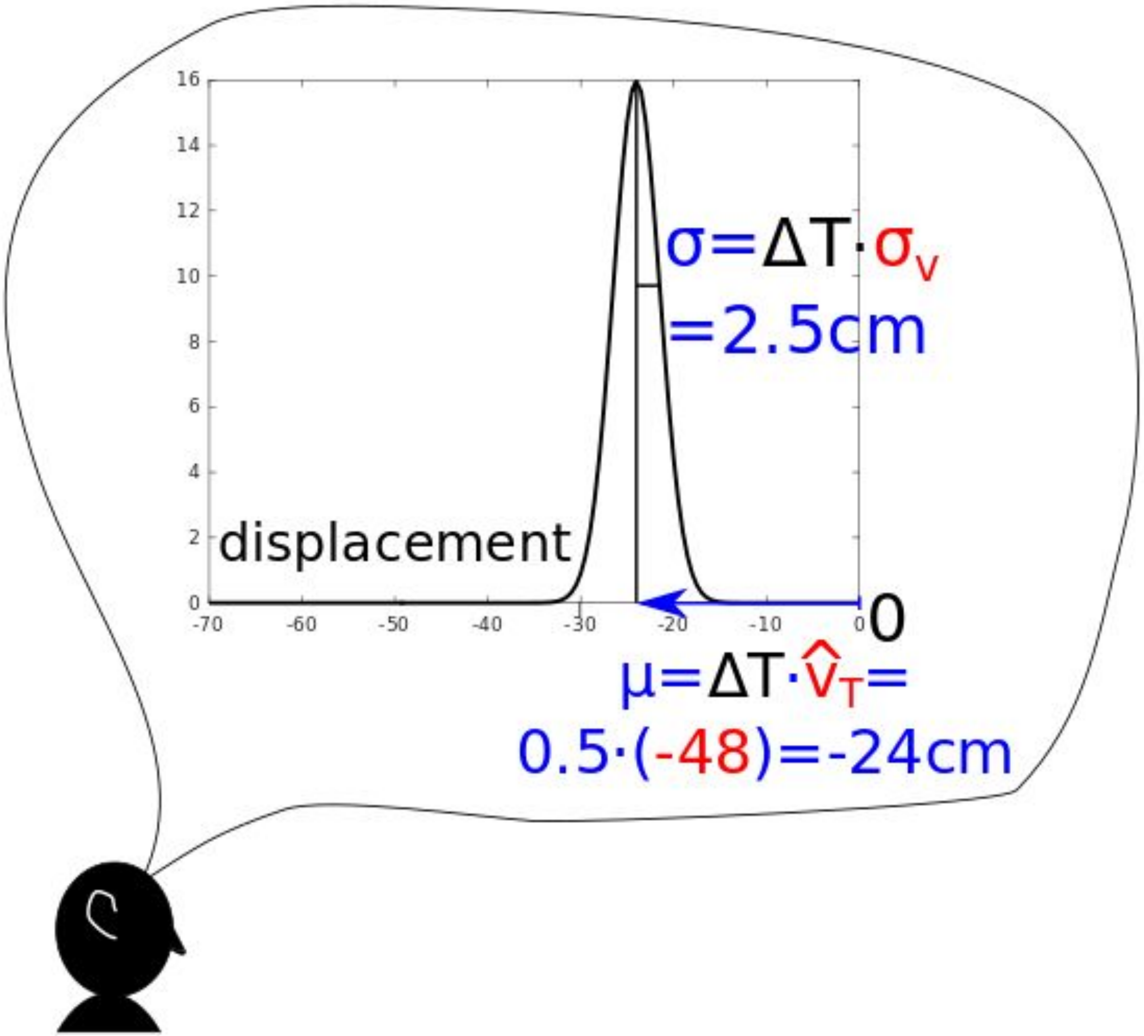

Using timestep ΔT=0.5 and the velocity estimation, object displacement distribution is calculated.

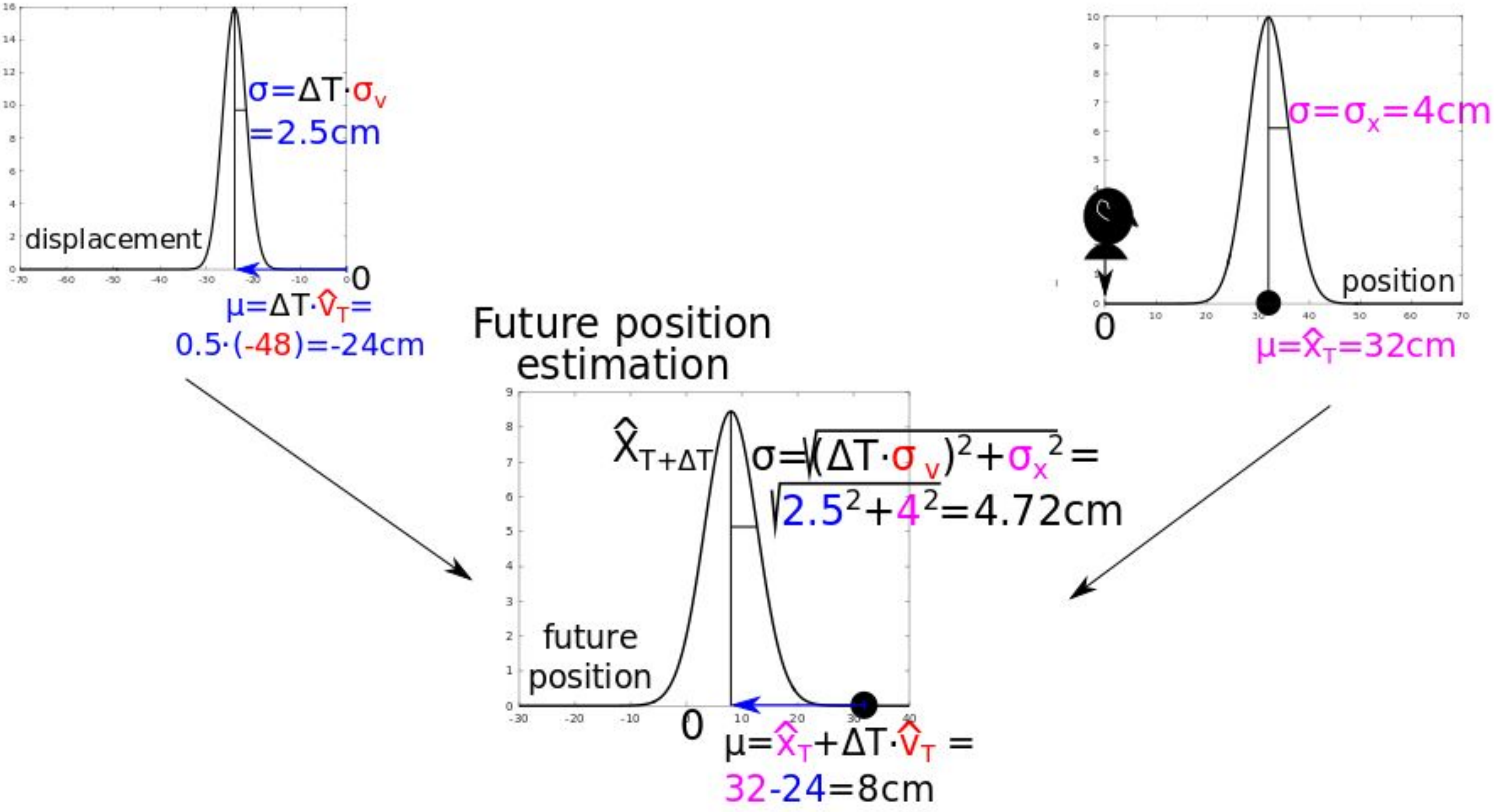

Merging the position and displacement estimations, future position distribution is estimated.

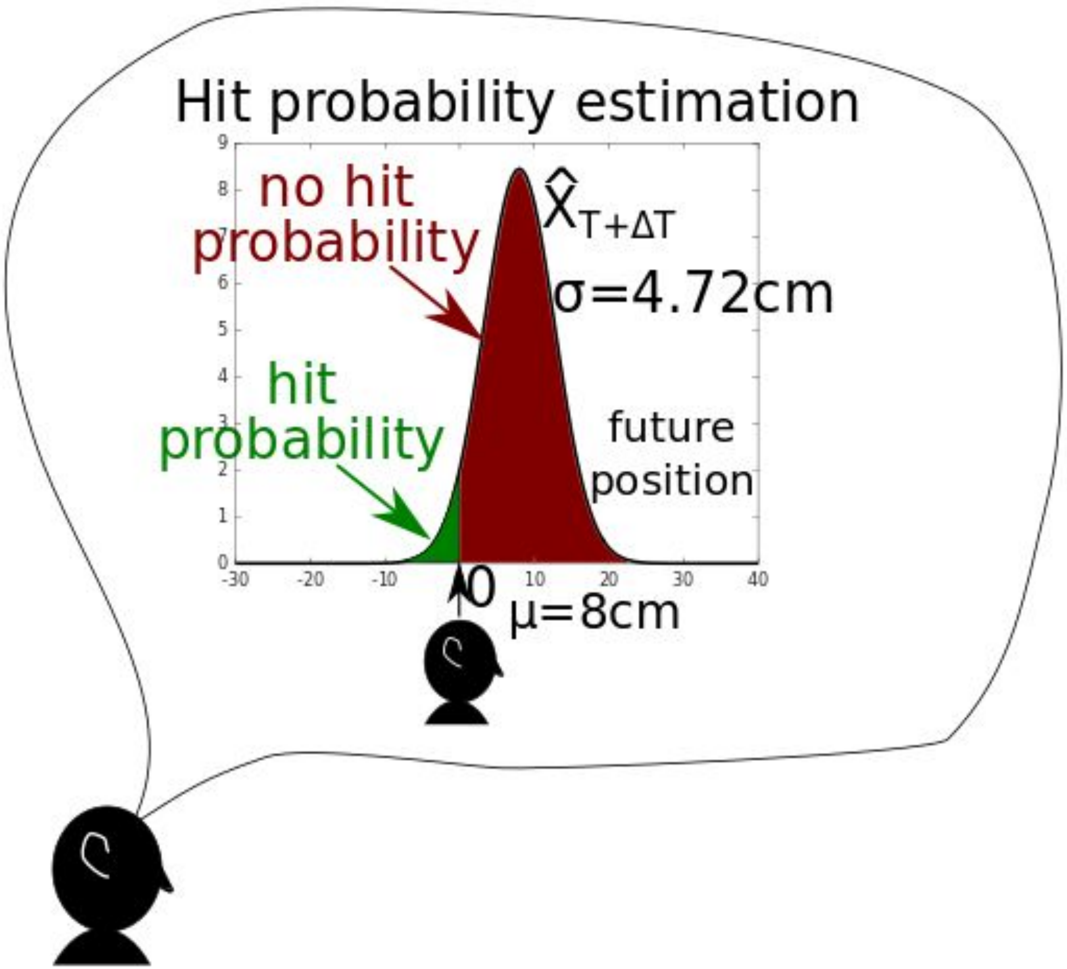

The future position distribution
is used for hit probability estimation.

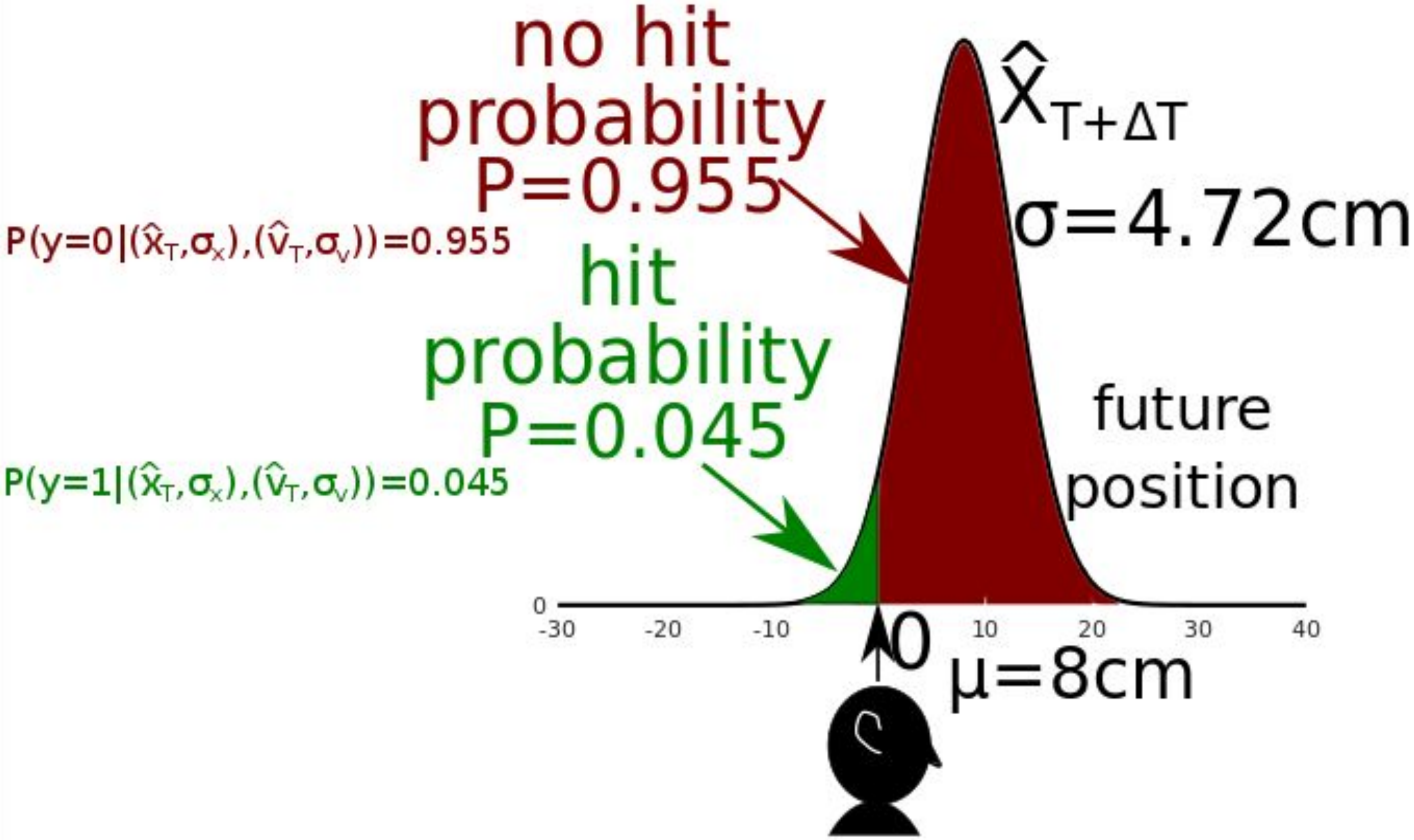

Hit probability estimation corresponds to the probability estimation that the object will be on the surface of the body or further in the space.

Hit probability estimation (previous step)

no hit probability: $P(y=0|(\hat{x}_T,\sigma_x),(\hat{v}_T,\sigma_v))=0.955$

hit probability: $P(y=1|(\hat{x}_T,\sigma_x),(\hat{v}_T,\sigma_v))=0.045$

Other parameters: $FN=5$, $FP=1$

The impact prediction y^*_{pred} is calculated as

$$y^*_{pred} = \arg \min_{y_{pred}\in[0,1]} L((\hat{x}_T,\sigma_x),(\hat{v}_T,\sigma_v),y_{pred})$$

where

$$\begin{aligned} L((\hat{x}_T,\sigma_x),(\hat{v}_T,\sigma_v),y_{pred}) &= P(y=1|(\hat{x}_T,\sigma_x),(\hat{v}_T,\sigma_v)) \cdot loss(y=1,y_{pred}) + \\ &\quad P(y=0|(\hat{x}_T,\sigma_x),(\hat{v}_T,\sigma_v)) \cdot loss(y=0,y_{pred}) = \\ P(y=1|(\hat{x}_T,\sigma_x),(\hat{v}_T,\sigma_v)) \cdot FN(1-y_{pred})^2 &+ P(y=0|(\hat{x}_T,\sigma_x),(\hat{v}_T,\sigma_v)) \cdot FP y^2_{pred} \\ &= 0.045 \cdot 5(1-y_{pred})^2 + 0.955 \cdot y^2_{pred} \end{aligned}$$

The impact prediction y^*_{pred} is calculated as

$$y^*_{pred} = \arg \min_{y_{pred} \in [0,1]} L((\hat{x}_T, \sigma_x), (\hat{v}_T, \sigma_v), y_{pred})$$

where

$$L((\hat{x}_T, \sigma_x), (\hat{v}_T, \sigma_v), y_{pred}) = 0.045 \cdot 5(1 - y_{pred})^2 + 0.955 \cdot y^2_{pred}$$

It can be solved numerically. The value of $L((\hat{x}_T, \sigma_x), (\hat{v}_T, \sigma_v), y_{pred})$ is calculated for $y_{pred} \in \{0, 0.05, 0.1, \ldots 1\}$:

$$L(\ldots, y_{pred} = 0) = 0.225$$

$$L(\ldots, y_{pred} = 0.05) = 0.205$$

$$\ldots$$

$$L(\ldots, y_{pred} = 0.2) = \mathbf{0.182}$$

$$\ldots$$

$$L(\ldots, y_{pred} = 1) = 0.955$$

The minimal value of L is reached for $y_{pred} = 0.2$. Therefore, the prediction is $y^*_{pred} = 0.2$.

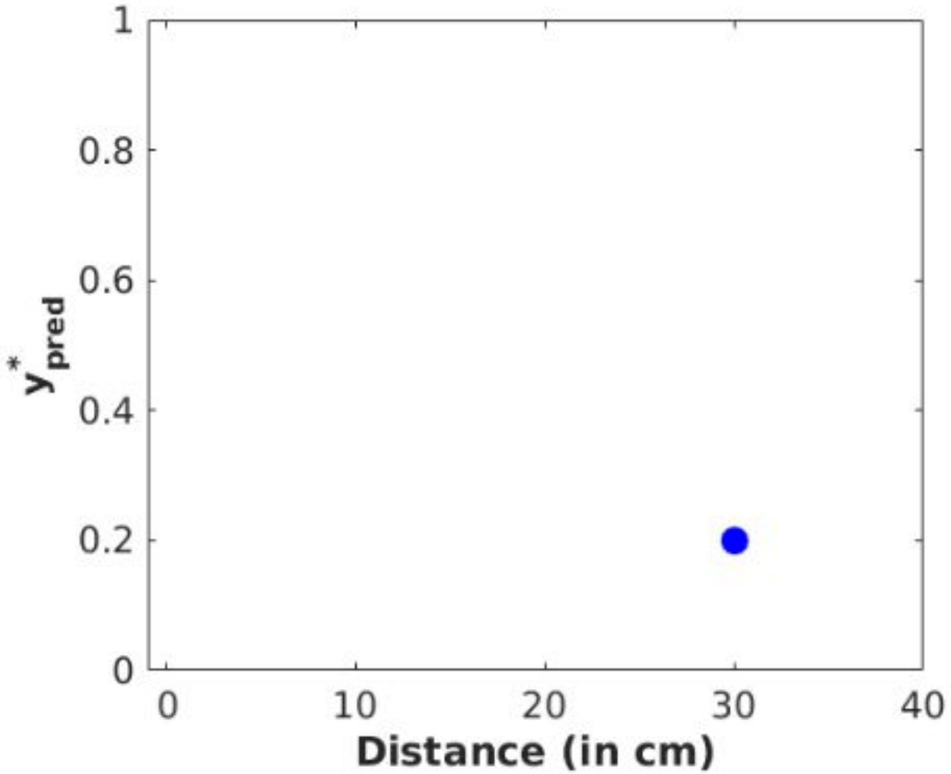

The calculated prediction for distance 30cm from the body is shown in the figure.

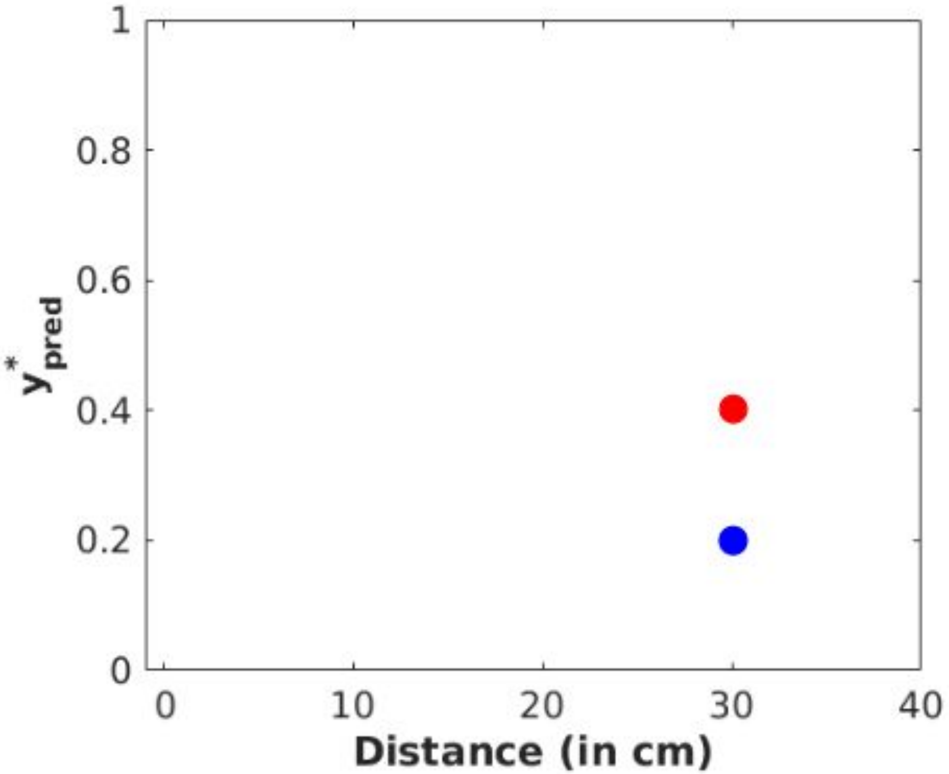

As the position and velocity estimation is stochastic, in the next repetition of the experiment, a different value can be predicted.
