# Supplementary material for: A normative model of peripersonal space encoding as performing impact prediction
Source: PLoS Comput Biol. 2022 Sep 14;18(9):e1010464. doi: 10.1371/journal.pcbi.1010464 (PMC9512250; doi:10.1371/journal.pcbi.1010464)
Supplement: S2 File — For a more interactive version see S1 File. (PDF) [file pcbi.1010464.s002.pdf]

S2 File

A detailed example of an impact prediction calculation. For a more interactive version see **S1 File**.

1. Position, velocity and displacement estimations
   (a) At time T, a stimulus has position $x_T = 30cm$ and velocity $v_T = -50cm/s$. Position and velocity estimations are disrupted by Gaussian noise with $\sigma_x = 4cm, \sigma_v = 5cm/s$, respectively.
   (b) Because of the noise, the point estimation of position is a sample $\hat{x}_T = 32cm = \textit{sample from } N(\mu = x_T = 30cm, \sigma = \sigma_x = 4cm)$ and velocity is a sample $\hat{v}_T = -48cm/s = \textit{sample from } N(\mu = v_T = -50cm/s, \sigma = \sigma_v = 5cm)$.
   (c) To include uncertainty, the position and velocity estimations are encoded as normal distributions $N(\mu = \hat{x}_T = 32cm, \sigma = \sigma_x = 4cm), N(\mu = \hat{v}_T = -48cm, \sigma = \sigma_v = 5cm)$, respectively.
   (d) Displacement estimation is calculated ($\Delta T = 0.5s$) $N(\mu = \Delta T \cdot \hat{v}_T = 0.5 \cdot (-48) = -24cm, \sigma = \Delta T \cdot \sigma_v = 0.5 \cdot 5 = 2.5cm)$.
2. Future position estimation calculation (sum of two random variables—the position and displacement estimations—with Gaussian distributions) $\hat{X}_{T+\Delta T} \sim N(\mu = \hat{x}_T + \Delta T \cdot \hat{v}_T = 32 - 24 = 8cm, \sigma = \sqrt{\sigma_x^2 + (\Delta T \cdot \sigma_v)^2} = \sqrt{4^2 + 2.5^2} = 4.72cm)$
3. Tactile impact probability estimation

$$P(y = 1|(\hat{x}_T, \sigma_x), (\hat{v}_T, \sigma_v)) = P(\hat{X}_{T+\Delta T} \leq 0) = 0.045,$$

$$P(y = 0|(\hat{x}_T, \sigma_x), (\hat{v}_T, \sigma_v)) = 1 - P(y = 1|(\hat{x}_T, \sigma_x), (\hat{v}_T, \sigma_v)) = 1 - 0.045 = 0.955$$

4. Optimal prediction selection ($FN = 5, FP = 1$)

$$y^*_{pred} = \arg \min_{y_{pred} \in [0,1]} L((\hat{x}_T, \sigma_x), (\hat{v}_T, \sigma_v), y_{pred}) \tag{1}$$

where $L((\hat{x}_T, \sigma_x), (\hat{v}_T, \sigma_v), y_{pred}) = P(y = 1|(\hat{x}_T, \sigma_x), (\hat{v}_T, \sigma_v)) \cdot loss(y = 1, y_{pred}) +$
$P(y = 0|(\hat{x}_T, \sigma_x), (\hat{v}_T, \sigma_v)) \cdot loss(y = 0, y_{pred}) =$
$P(y = 1|(\hat{x}_T, \sigma_x), (\hat{v}_T, \sigma_v)) \cdot FN(1 - y_{pred})^2 + P(y = 0|(\hat{x}_T, \sigma_x), (\hat{v}_T, \sigma_v)) \cdot FPy^2_{pred}$
$= 0.045 \cdot 5(1 - y_{pred})^2 + 0.955 \cdot y^2_{pred}$

It can be solved numerically. The value of $L((\hat{x}_T, \sigma_x), (\hat{v}_T, \sigma_v), y_{pred})$ is calculated for $y_{pred} \in \{0, 0.05, 0.1, \ldots 1\}$:

$$L(\ldots, y_{pred} = 0) = 0.225$$

$$L(\dots, y_{pred} = 0.05) = 0.205$$

$$\dots$$

$$L(\dots, y_{pred} = 0.2) = \mathbf{0.182}$$

$$\dots$$

$$L(\dots, y_{pred} = 1) = 0.955$$

The minimal value of L is reached for $y_{pred} = 0.2$. Therefore, the prediction is $y^*_{pred} = 0.2$.
